# China’s deserts greening and response to climate variability and human activities

**DOI:** 10.1371/journal.pone.0256462

**Published:** 2021-08-30

**Authors:** Xiaoyu Liu, Liangjie Xin

**Affiliations:** 1 Key Laboratory of Land Surface Pattern and Simulation, Institute of Geographic Sciences and Natural Resources Research, Chinese Academy of Science, Beijing, China; 2 College of Resources and Environment, University of Chinese Academy of Sciences, Beijing, China; United Nations University Institute for Natural Resources in Africa, GHANA

## Abstract

Vegetation, which is a good indicator of the impacts of climate variability and human activities, can reflect desert ecosystem dynamics. To reveal the vegetation variations in China’s deserts, trends in the monthly, seasonal, and annual normalized difference vegetation index (NDVI) from 2000 to 2017 were measured both temporally and spatially by the Theil-Sen estimator and Mann-Kendall test. Additionally, correlation coefficients and residual analysis were employed to evaluate the correlations between the NDVI and climatic factors and to distinguish the impacts of climate variability and human activities. The results showed that China’s deserts underwent greening. The annual NDVI showed a significant increasing trend at a rate of 0.0018/yr, with values of 0.094 in 2000 and 0.126 in 2017. Significant increasing trends in NDVI were observed in all four seasons. The NDVI were higher in summer and autumn than in spring and winter. Both the monthly NDVI and its trends showed an inverted U-shaped curve during the year. Spatially, the greening trends were mainly distributed on the southern edge of the Gurbantunggut Desert, in the northwestern part of the Taklimakan Desert, and in the Kubuqi Desert. The correlations between the NDVI and climatic factors at the monthly and seasonal scales were stronger than those at the annual scale. Temperature and precipitation had positive effects on NDVI at the monthly and seasonal scales, but only precipitation had a positive effect at the annual scale. Human activities, especially oasis expansion and sand stabilization measures, were two major causes of large increasing areas of desert greening in China indicated by the NDVI.

## Introduction

Deserts are one of the major land-cover types, covering approximately one-third of the terrestrial area of the earth [1]. Due to topography, atmospheric circulation, latitude, ocean currents, etc., deserts, as a product of arid climatic conditions, are mostly located on the western coasts of continents near 30° north and south latitude [2,3]. There is still no universally accepted common or technical definition for deserts, only distinguished by characteristics (e.g., climate, weather, and hydrology) [4]. In addition, deserts are dynamic, and their boundaries are gradual and not clearly defined. In China, deserts are mainly distributed in the northwest, which is affected by both East Asian monsoons and global westerlies, and the climate variability that occurs within a year is critically influenced by the interplay of monsoonal circulation between summer and winter [5].

As a potential result of global warming [6], desert ecosystems as one of the fragile ecosystems are changing dynamically [3]. To clarify the changes of desert ecosystems can help to understand their ecological balance, and to take effective measures in time. It is important to strengthen ecological construction and take effective countermeasures in time. Due to the sensitivity to climate variability and good performance in arid areas [7–9], vegetation can be an ideal indicator to detect changes in desert ecosystems dynamics [10]. The normalized difference vegetation index (NDVI), which provides information on vegetation conditions, can identify low biomass and an increase in vegetation cover than other indexes [11], so it has become the most widely used index in studies on vegetation dynamics [12,13]. In recent decades, several studies have focused on NDVI variations in arid and semiarid regions [14–16]. Their results showed that the NDVI showed greening trends at the annual scale in several regions, such as the Gurbantunggut Desert, which showed an increasing trend that fluctuated from 1981–2003 [17]. At the same time, the NDVI for Central Asia significantly increased, with a value of 11.35% [18]. In global semiarid areas, the average NDVI rise from 1981 to 2007 by about 0.015 NDVI units [19]. Seasonal differences were seen in the global NDVI, for example, the upward trend of NDVI in spring, summer, and autumn was observed in some mid- and high-latitude regions of the Northern Hemisphere, and the downward trend of NDVI in summer and autumn was observed in some arid and semi-arid regions of the Southern Hemisphere [20]. Variations in the NDVI that occur within a year can be observed at the monthly scale. For instance, the monthly mean NDVI increased between January and August and decreased in December in Nigeria [15]. However, the monthly mean NDVI in Central Asia is highest in January and December [21].

Variations in the NDVI are influenced by multiple factors, which can be divided into environmental and anthropogenic factors [22–24]. Among various environmental factors, variations in temperature and precipitation, which are forms of climate variability, are considered to be the two most influential factors [25–27]. In most cases, the effect of temperature is positive, but sometimes it has a negative effect. For example, the NDVI is positively associated with the temperature when energy is limited to vegetation growth in higher latitudes and higher altitudes in the North American continent, especially at the beginning of the season, but usually in the mid-season the relationship is negative for lower latitudes when water is the limit of vegetation growth [28]. Temperature has a positive correlation with NDVI at both ends of the growing seasons but a weak negative correlation in the middle of the growing season in the central Great Plains [29]. Precipitation positively influences the NDVI, especially in arid and semiarid regions that lack water [30–32]. The impact of climate variability also has seasonal differences, as temperature is the dominant factor in spring that lowers the NDVI in inner Asia, which is an environment appropriate for vegetation growth and causes an increase in the melting of winter snow and ice, thereby increasing moisture in the spring [33,34]. Sufficient rainfall during summer benefits the growth of vegetation in arid areas more [35]. The effect of climate has a time lag that is distributed differently in different regions. For example, there was a 3-month lag between the NDVI and temperature at the biome scale in China [36], and there was a 2-year lag in temperature at the global scale [37]. Human activities such as cropland and forest expansion in China and India have increased their relative areas by approximately 25% and 6.8%, respectively, and have played a critical role in global greening [22]. However, a dramatic decline in NDVI was observed in the Yangtze River and Pearl River deltas due to rapid urbanization [36].

The NDVI is an effective indicator of conditions affecting vegetation growth and can reflect the impacts of environmental and anthropogenic factors. Previous studies have paid more attention to NDVI dynamics and influential factors in different regions (e.g., Hungary [38], Central Asia [21], and Canada [39]), as well as different land cover types (e.g., grassland [40], forests [41], and cropland [42]). By contrast, few studies have considered NDVI variations and driving factors at both the interannual and intra-annual scales in regions with low vegetation cover, especially arid zones and deserts, which are more sensitive to outside factors.

Combined with existing studies, it has been found that vegetation in most arid zones around the world has shown a gradual recovery trend recently [8,43]. As a country with vast desert areas, China has been under the attack of wind and sand for years, the government attached great importance to it and made great practices in desert control, especially since the 21^st^ century, ecological protection has been highly emphasized in China. However, the detailed studies on the vegetation dynamics of all deserts in China and the extent to which they are affected by climatic factors and human activities are still lacking.

This study aims to analyze NDVI variations and the influential factors in China’s deserts from 2000 to 2017. Specifically, this study seeks to (1) use monthly, seasonal, and annual data to explore trends in the NDVI and climatic factors temporally and spatially; (2) analyze the correlations between the NDVI and temperature and precipitation at the monthly, seasonal, and annual scales to better understand the relationships between the NDVI and climatic factors and to compare their influence at different time scales; and (3) use residual analysis to distinguish greening regions caused by human activities from 2000 to 2017.

## Materials and methods

### Study area

**Fig 1:**
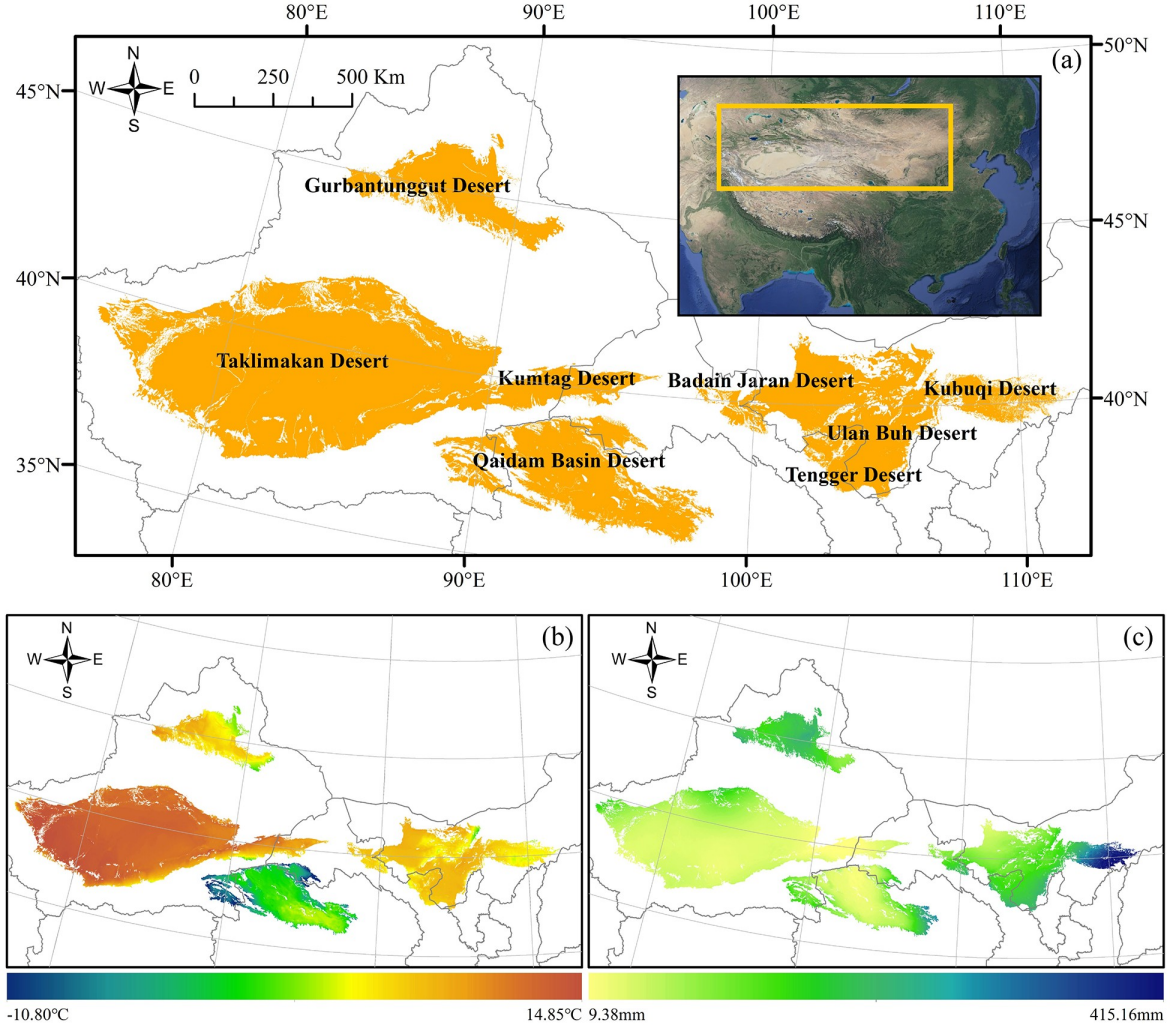
Spatial distribution of deserts in China(a), and mean annual temperature(b) and precipitation(c) of China’s deserts from 2000 to 2017.

Deserts in China are widely distributed in the north, especially northwestern China, which is located between 75°-115°E and 35°-47°N, with an area of approximately 0.98 million km^2^. Most part of the deserts is distributed in arid areas, and the remaining part is distributed in semi-arid areas. The annual average temperature of China’s deserts ranges from -10°C to 15°C, and the annual accumulated precipitation in most of the areas is less than 200 mm and mostly distributed in summer. Eight deserts are located in the study area: the Gurbantunggut Desert, Taklimakan Desert, Kumtag Desert, Qaidam Basin Desert, Badain Jaran Desert, Tengger Desert, Ulan Buh Desert, and Kubuqi Desert (Fig 1(a)).

### Data sources

#### Distribution of deserts

The desert distribution data used in this study were mainly obtained from the 1:100000 desert (sand) distribution dataset in China [44]; the data were interpreted, extracted, and edited from Thematic Mapper (TM) digital images and the China land-use map for 2000. The range of deserts was corrected by using a topographic map of China and the map of desert distribution at 1:2000000 in China [45].

#### NDVI data

In this study, the NDVI from MOD13A2 was used as an indicator of vegetation activity. The NDVI dataset was sourced from the MODIS (Moderate Resolution Imaging Spectroradiometer) vegetation index product data on the NASA website (https://lpdaac.usgs.gov/products/mod13a2v006/) [46] and had already been corrected for the effect of atmospheric gases, thin cirrus clouds, and aerosols, with a spatial resolution of 1 km and a temporal resolution of 16 days. A total of 411 images were used for the period between December 2017 and February 2000. Data processing, such as image merging, map projection transformation, resampling, and masking by desert areas, was performed on the original downloaded NDVI data. The maximum value composite (MVC) method was applied to calculate the monthly and annual NDVI data for each pixel to reduce the atmospheric effects of clouds and aerosols [47–49]. The mean values for spring (March-May), summer (June-August), autumn (September-November), and winter (January, February, and December) were calculated separately to truly reflect seasonal differences in the NDVI. Otherwise, the maximum values of the NDVI for spring, summer, and autumn would be very similar, but a large gap would appear for winter.

#### Climate data

Data on the monthly mean temperature and monthly accumulated precipitation from 2000 to 2017 at a 1 km spatial resolution were obtained from the National Tibetan Plateau Data Center (http://data.tpdc.ac.cn) [50–52]. The original climate data were merged, projection transformed, masked, and registered to be consistent with the NDVI data. The seasonal and annual mean temperature and accumulated precipitation were calculated from monthly values to aid the analysis. Multi-year averages of temperature (Fig 1(b)) and precipitation (Fig 1(c)) from 2000 to 2017 were also calculated for understanding the climatic conditions of the study area.

Both the NDVI raster data and climate raster data were processed and analyzed by using ArcGIS10.5, MATLAB R2016a, and Python 3.7.

### Methods

#### Theil-Sen estimator

The Theil-Sen estimator calculates the slope estimate as the median of the single-point slope and the intercept as the median of the single-point intercept. The Theil-Sen estimator can effectively eliminate the influence of outliers on the estimation results (Eq 1) [53,54]. In contrast to ordinary least squares used for linear regression, the Theil-Sen estimator is an unbiased estimate of a linear trend and is more robust; therefore, it has been widely used for estimating the slope of the NDVI [55–58].

$$slope=Median\left(\frac{NDVI_{j}-NDVI_{i}}{j-i}\right),\forall j>i$$ (1)

where *NDVI*_*i*_ and *NDVI*_*j*_ are the NDVI values at times *i* and *j*, respectively. The Theil-Sen estimator was used to calculate the trend for each pixel; in general, *slope* >0 indicates a positive trend in the NDVI value of the pixel during the period, and vice versa.

#### Mann-Kendall test

The Mann-Kendall test, which is a nonparametric test, was applied to evaluate the significance of the trend of the Theil-Sen estimator [59,60]. And it has been widely used in analyses of NDVI trends [39,61–63]. The Mann-Kendall test was computed using Eqs (2–4).

$$S=\overset{n-1}{\underset{i=1}{\sum }}\overset{n}{\underset{j=i+1}{\sum }}sign\left(NDVI_{j}-NDVI_{i}\right)$$ (2)

$$sign\left(NDVI_{j}-NDVI_{i}\right)=\left\{\begin{array}{c} 1\;\;\;\;\;\;\;\;\;\;\;(NDVI_{j}-NDVI_{i}>0)\\ 0\;\;\;\;\;\;\;\;\;\;(NDVI_{j}-NDVI_{i}=0)\\ -1\;\;\;\;\;\;\;(NDVI_{j}-NDVI_{i}<0) \end{array}\right.$$ (3)

$$Z=\left\{\begin{array}{c} \frac{S-1}{\sqrt{Var\left(S\right)}}\;\;\;\;\;\;\;\;\;\;\;(S>0)\\ \;\;\;\;0\;\;\;\;\;\;\;\;\;\;\;\;\;\;\;\left(S=0\right)\\ \frac{S+1}{\sqrt{Var\left(S\right)}}\;\;\;\;\;\;\;\;\;\;\;(S<0) \end{array}\right.$$ (4)

where *n* is the length of the time-series data (in this study, *n* = 18), *S* represents the Mann-Kendall statistic, and *Z* is a standardized statistic that follows a standard normal distribution. |*Z*| > 1.96 indicates that the time-series data change significantly at the 5% level.

Combined with the Mann-Kendall statistic and the Theil-Sen estimator, a pixel shows a greening trend when *slope >* 0 and |*Z*| > 1.96, the pixel shows a browning trend when *slope <* 0 and |*Z*| > 1.96, and the pixel does not show a significant change when |*Z*| ≤ 1.96.

#### Correlation analysis of the NDVI and climatic factors

The Pearson correlation coefficient (r) was commonly used in previous studies to evaluate the relationships between vegetation dynamics and climatic factors [43,64,65]. In this study, this method was applied to calculate correlations between monthly, seasonal, and annual NDVI and climatic factors (i.e., temperature and precipitation). P-values were used to indicate the significance of the correlations.

#### Residual analysis

NDVI variations are jointly affected by climate variability and human activities [64,66,67]. However, the impact of human activities on the NDVI of deserts is difficult to assess by using only one indicator or several indicators. The residual analysis method can separate the influence of climate factors and human activities with the help of multiple linear regression (Eqs 5 and 6), so it is widely used to identify the impact of human activities [23,68].

$${NDVI}_{pre,n}\;=\;a*{TMP}_{n}+b*{PRE}_{n}+c$$ (5)

$${NDVI}_{human}\;=\;{NDVI}_{obs}-{NDVI}_{pre,n}$$ (6)

where *NDVI*_*pre*,*n*_ is the value of the NDVI predicted by temperature and precipitation in year *n*. *TMP*_*n*_ and *PRE*_*n*_ are annual mean temperature and annual accumulated precipitation in year *n*. *a* and *b* are the coefficients of temperature and precipitation, respectively, calculated by multiple linear regression to obtain. *c* represents the intercept of the multiple linear regression. *NDVI*_*human*_ represents the variations in NDVI affected by human activities. *NDVI*_*obs*_ represents observed NDVI.

## Results

### Temporal variation of NDVI and climatic factors

**Fig 2:**
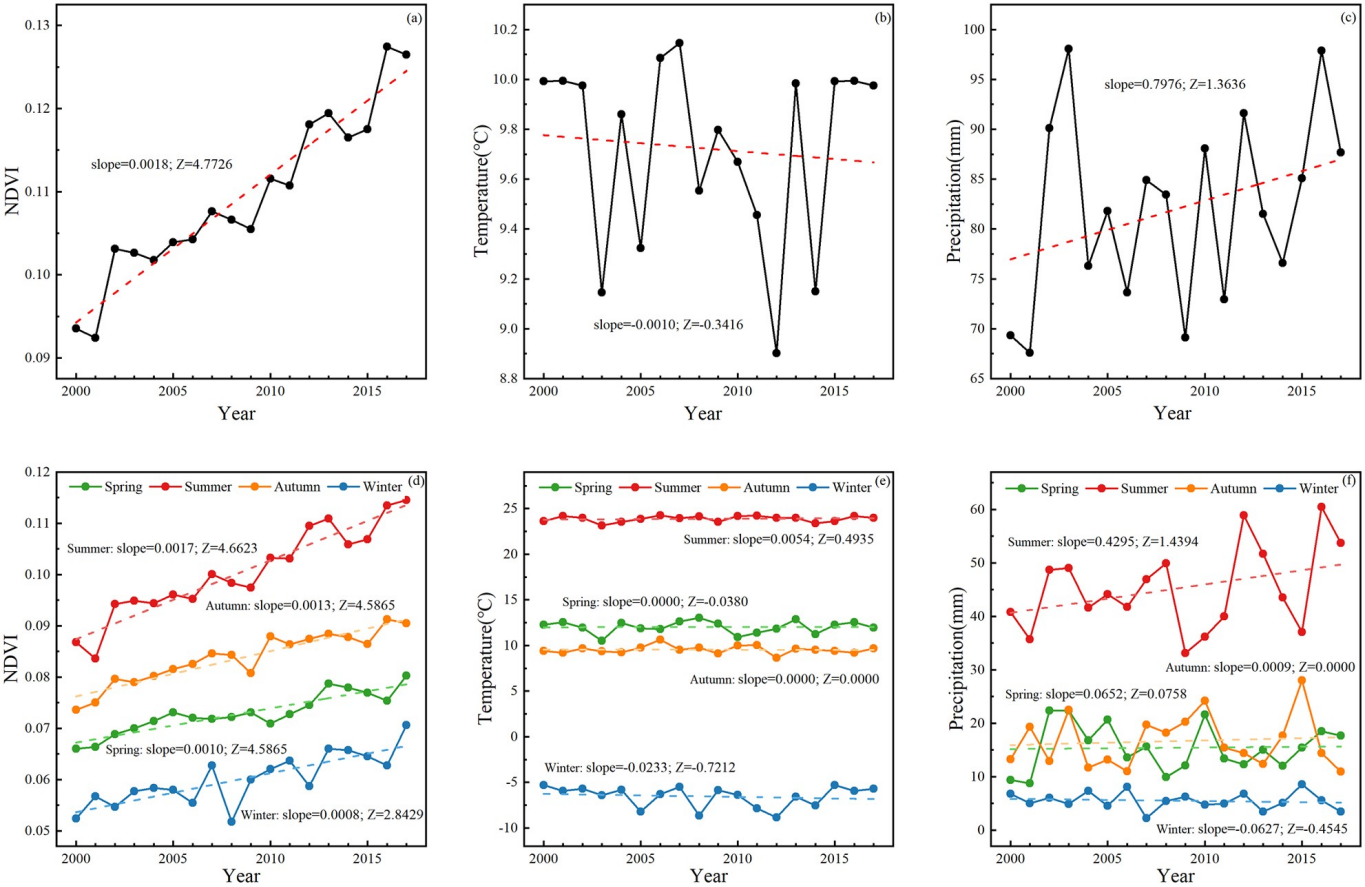
Variations and trends in annual precipitation (a), temperature (b), and NDVI (c), and seasonal NDVI (d), temperature (e), and precipitation (f) in desert areas from 2000 to 2017.

The mean values of the monthly, seasonal, and annual NDVI, temperature, and precipitation of the whole study area were calculated for use in the analysis of the temporal variations, and the trends were identified by using the Theil-Sen estimator and Mann-Kendall test. According to the results (Fig 2a-2c), the annual NDVI showed a significant increasing trend at a rate of 0.0018/yr (|*Z*| > 1.96), up from 0.094 in 2000 to 0.126 in 2017. However, temperature and precipitation showed a slight decreasing trend (-0.0010/yr) and an increasing trend (0.7976/yr), respectively, and both fluctuated greatly and showed insignificant trends.

**Fig 3:**
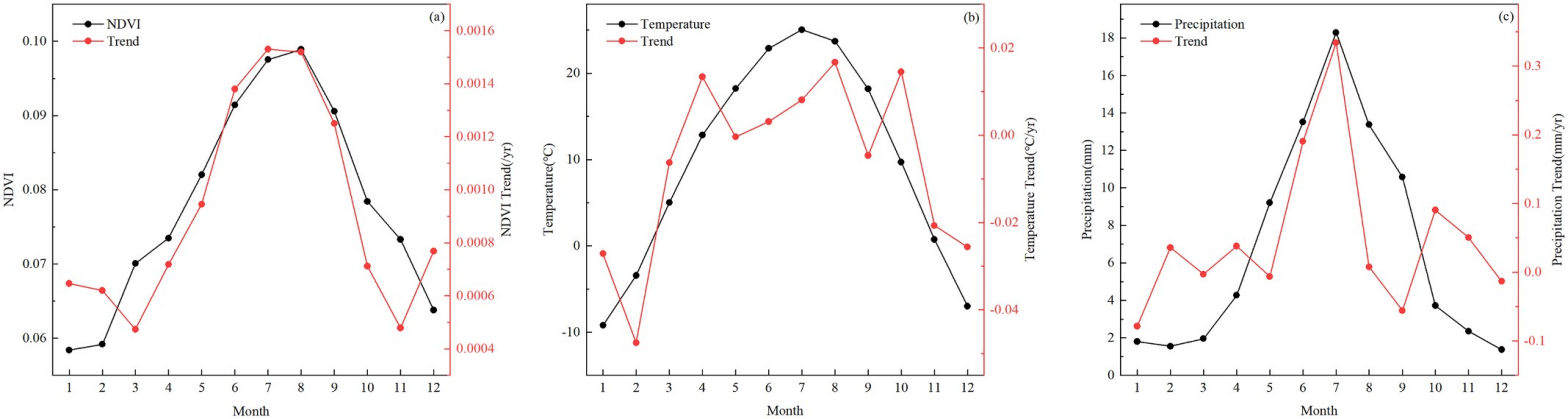
Mean values (black lines) and trends (red lines) of the monthly NDVI (a), temperature (b), and precipitation (c) in desert areas from 2000 to 2017.

The seasonal differences in the NDVI were obvious. The mean values of the NDVI from 2000 to 2017 for each season were as follows: summer (0.1005)>autumn (0.0837)>spring (0.0729)>winter (0.0601). The seasonal temperature showed a different pattern and was higher in spring than in autumn. Because of the large fluctuations of seasonal precipitation in the time series, the variation curves for spring and autumn precipitation were staggered. With respect to the trends, the seasonal NDVI (Fig 2(d)) showed a significant increasing trend in all four seasons (|*Z*| > 1.96), with summer showing the highest rate of increase at 0.0017/yr, followed by autumn (0.0013/yr) and spring (0.0010/yr), and winter showing the lowest rate of increase (0.0008/yr). Seasonal temperature and precipitation did not show significant fluctuation trends. The temperature showed slight fluctuations, with increasing trends in summer (0.0054°C/yr), and decreasing trends in winter (-0.0233°C/yr). There were greater fluctuations in seasonal precipitation, especially in summer, with increasing trends in summer (0.4295 mm/yr) and a decreasing trend in winter (-0.0627 mm/yr). The data showed monthly NDVI variations (Fig 3). The mean values of the monthly NDVI from 2000 to 2017 showed an inverted U-shaped curve, which was the lowest in January (0.0539) and the highest in August (0.0989). The variations in monthly temperature and precipitation were the same as the NDVI variations with one exception: the maximum values of both temperature and precipitation occurred in July. The trend of the NDVI over 12 months was positive, implying that the NDVI increased throughout the year during the study period.

### Spatial differences in greening and browning trends

#### Greening and browning trends indicated by the annual NDVI

**Fig 4:**
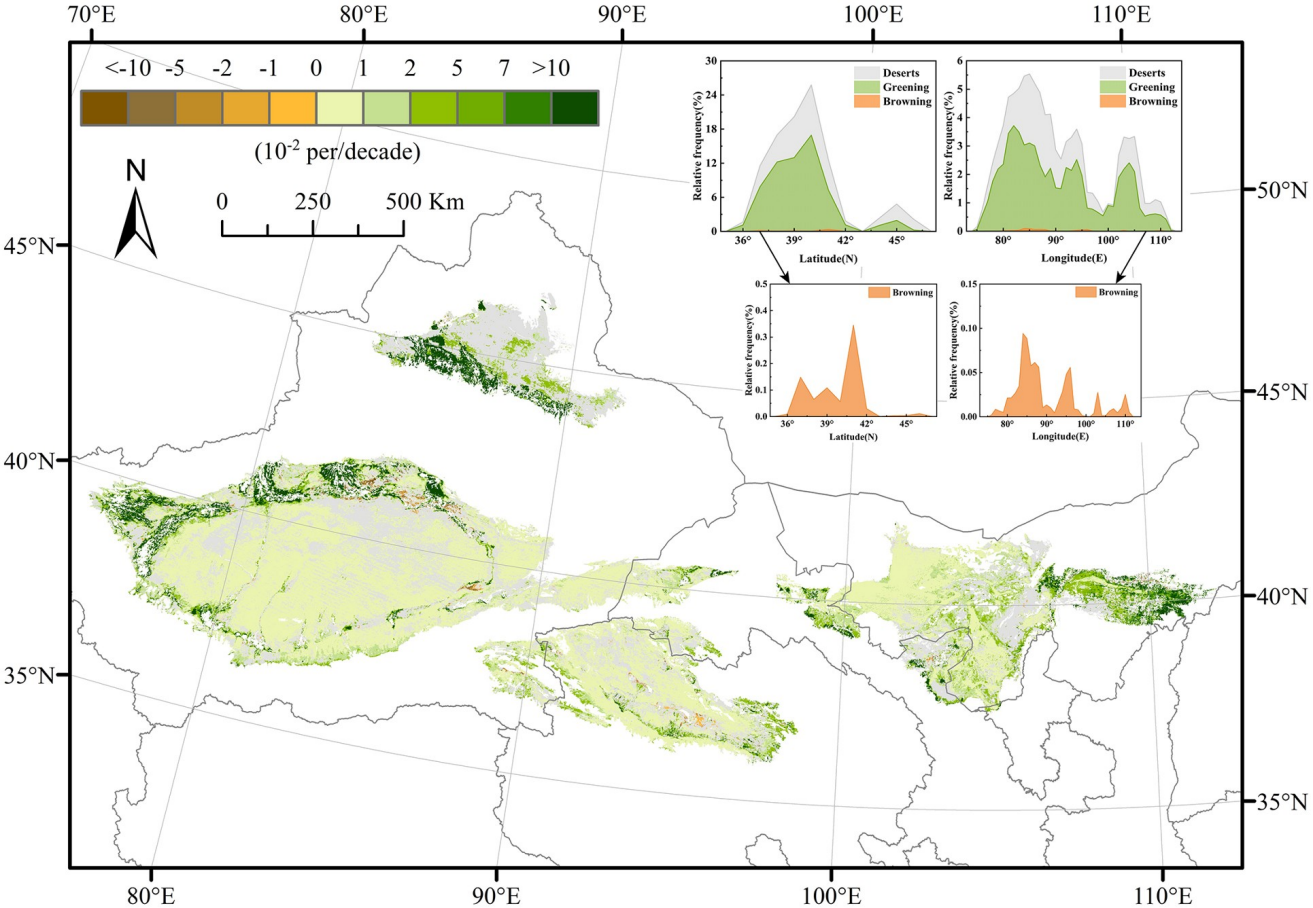
Spatial distribution of greening and browning trends and statistical results based on the longitude and latitude of China’s desert areas from 2000 to 2017. Gray areas represent desert regions with no significant trends at the 5% confidence level. The green and brown areas indicate that the pixel has a significant greening and browning trend, respectively.

On the annual scale, the deserts showed greening trends spatially (Fig 4), with 62.8% of the area showing greening trends, mainly on the desert margins, especially on the southern edge of the Gurbantunggut Desert, in the northwestern part of the Taklimakan Desert, and in the Kubuqi Desert. Only 0.78% of the area showed browning trends, distributed in the northeastern Taklimakan Desert and the eastern Qaidam Basin Desert.

Fig 4 also shows the relative frequency distributions of the greening and browning trends over longitude and latitude. The area with greening trends was positively correlated with the desert area. Although the area with browning trends was very small, it was distributed over longitudes and latitudes with large desert areas.

The NDVI trends differed for different deserts (Table 1). The proportion of greening in the Badain Jaran Desert was the highest among the deserts at 88.26%, which increased significantly, followed by the Kumtag Desert (75.26%) and Tengger Desert (70.07%). While the proportion of greening in the Gurbantunggut Desert was only 31.22%, the lowest of the deserts. The Qaidam Basin Desert (1.25%) showed the highest proportion of browning, followed by the Taklimakan Desert (0.84%) and the Kubuqi Desert (0.67%).

**Table 1:** The NDVI trends in different deserts.

Desert	Significant Increase (%)	Increase but not significant (%)	Significant decrease (%)	Decrease but not significant (%)
**Gurbantunggut Desert**	31.22	62.50	0.17	6.12
**Kumtag Desert**	75.26	23.30	0.07	1.38
**Qaidam Basin Desert**	63.60	30.46	1.25	4.68
**Kubuqi Desert**	60.31	33.67	0.67	5.35
**Badain Jaran Desert**	88.26	11.14	0.05	0.55
**Ulan Buh Desert**	40.41	50.40	0.30	8.89
**Tengger Desert**	70.07	27.67	0.11	2.15
**Taklimakan Desert**	65.82	29.69	0.84	3.65

#### Greening and browning trends indicated by the seasonal NDVI

**Fig 5:**
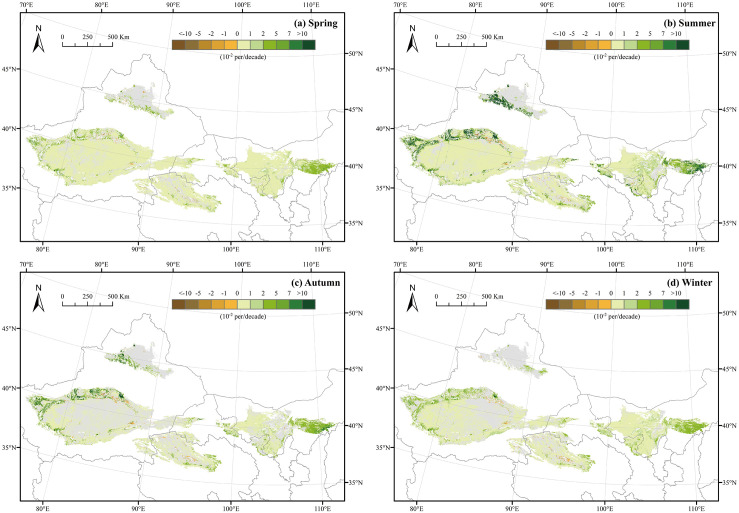
Spatial distribution of greening and browning trends for spring (a), summer (b), autumn (c), and winter (d) in China’s desert areas from 2000 to 2017. Gray areas represent desert regions with no significant trends at the 5% confidence level. The green and brown areas indicate that the pixel has a significant greening and browning trend, respectively.

The greening and browning trends were calculated by the Theil-Sen estimator and Mann-Kendall test using the seasonal NDVI in desert areas from 2000 to 2017 (Fig 5) and the statistics are shown in Table 2. The spatial distribution of the greening and browning trends had obvious seasonal differences. In spring and winter, there was little spatial difference among the deserts, and the areas showed approximately the same degree of greening. The proportion of greening areas in spring was 73.05%, the highest among the seasons. However, the degree of the greening of NDVI in autumn was higher than that in spring, and the greening trends increased on the southern edge of the Gurbantunggut Desert, in the northwestern part of the Taklimakan Desert, and in the Kubuqi Desert. The spatial distribution of the NDVI trends in summer was the most similar to the spatial distribution of the annual NDVI trends; this may have occurred because the annual NDVI was calculated by the MVC method, while the maximum values of NDVI in most regions occurred in summer.

**Table 2:** Proportion of the area for seasonal NDVI trends.

Season	Significant Increase (%)	Increase but not significant (%)	Significant decrease (%)	Decrease but not significant (%)
**Spring**	73.05	22.30	0.76	3.89
**Summer**	69.37	25.95	0.96	3.72
**Autumn**	44.93	46.02	1.14	7.91
**Winter**	56.49	35.34	0.86	7.30

### Correlations between the NDVI and climatic factors

The Pearson correlation coefficients between monthly, seasonal, and annual NDVI and climatic factors were calculated to measure the effect of climate variability on NDVI in deserts and to compare the impact of different time scales on the correlations, as presented in Table 3.

**Table 3:** The Pearson correlation coefficients of the climatic factors and NDVI at different time scales.

Scale	Temperature	Precipitation
**Annual**	-0.087	0.544*
**Seasonal**	0.865**	0.866**
**Monthly**	0.869**	0.796**

* Significant at level 0.10;

** significant at level 0.05.

Comparing the differences in the effects of climatic factors on NDVI at different scales, it was found that the correlations between climatic factors and NDVI were significant at seasonal and monthly scales, while only precipitation was significantly correlated with NDVI at annual scales. This suggested that climate variability was the main cause of monthly and seasonal variation in NDVI, while only precipitation had some influence on NDVI variation at the interannual scale.

#### Lagged effect of climatic factors on NDVI

Considering the possible lag in the effect of temperature and precipitation on the NDVI, the Pearson correlation coefficients for the correlations between the current NDVI of each month and the climatic factors of the current month and previous months were calculated (Table 4).

**Table 4:** The Pearson correlation coefficient for the relationship between the monthly NDVI and temperature and precipitation for different time lags.

Time lag	Temperature	Precipitation
**0**	0.796**	0.869**
**1**	0.764**	0.864**
**2**	0.532**	0.622**
**3**	0.115	0.226**
**4**	-0.286**	-0.227**

0 indicates the correlation coefficients are calculated by the NDVI and climatic factors in the same month, 1 indicates the correlation coefficients are calculated by the NDVI in the current month and climatic factors in the previous month, etc.

** Significant at level 0.05.

The results showed that temperature and precipitation had no lagged effect on the monthly NDVI because the largest correlation coefficients for the correlations between the NDVI and temperature and precipitation in the same month were 0.796 and 0.869, respectively, and the correlation decreased as the number of lagged months increased. When the lagged months increased to 4 months, NDVI started to be negatively correlated with temperature and precipitation.

#### Spatial differences in correlations

To further compare the differences in the impacts of climate variability on NDVI at different scales spatially, the Pearson correlation coefficients for the relationship between monthly and annual NDVI and temperature and precipitation from 2000 to 2017 were calculated for each pixel, and the proportion of the area of the correlation coefficient is shown in Table 5.

**Table 5:** Proportion of the area of the correlation coefficients for the relationships between the monthly and annual NDVI and temperature and precipitation.

Scale	Correlation level	Temperature	Precipitation
**Monthly**	Significant positive (%)	71.04	68.19
	Positive but not significant (%)	23.11	15.09
	Significant negative (%)	2.22	5.93
	Negative but not significant (%)	3.63	10.79
**Annual**	Significant positive (%)	0.50	14.99
	Positive but not significant (%)	38.35	69.97
	Significant negative (%)	3.98	0.15
	Negative but not significant (%)	57.17	14.89

“Significant positive” denotes pixels r>0 and p<0.05, “Positive but not significant” denotes pixels r>0 and p>0.05, “Significant negative” denotes pixels r<0 and p<0.05, and “Negative but not significant” denotes pixels r<0 and p>0.05.

**Fig 6:**
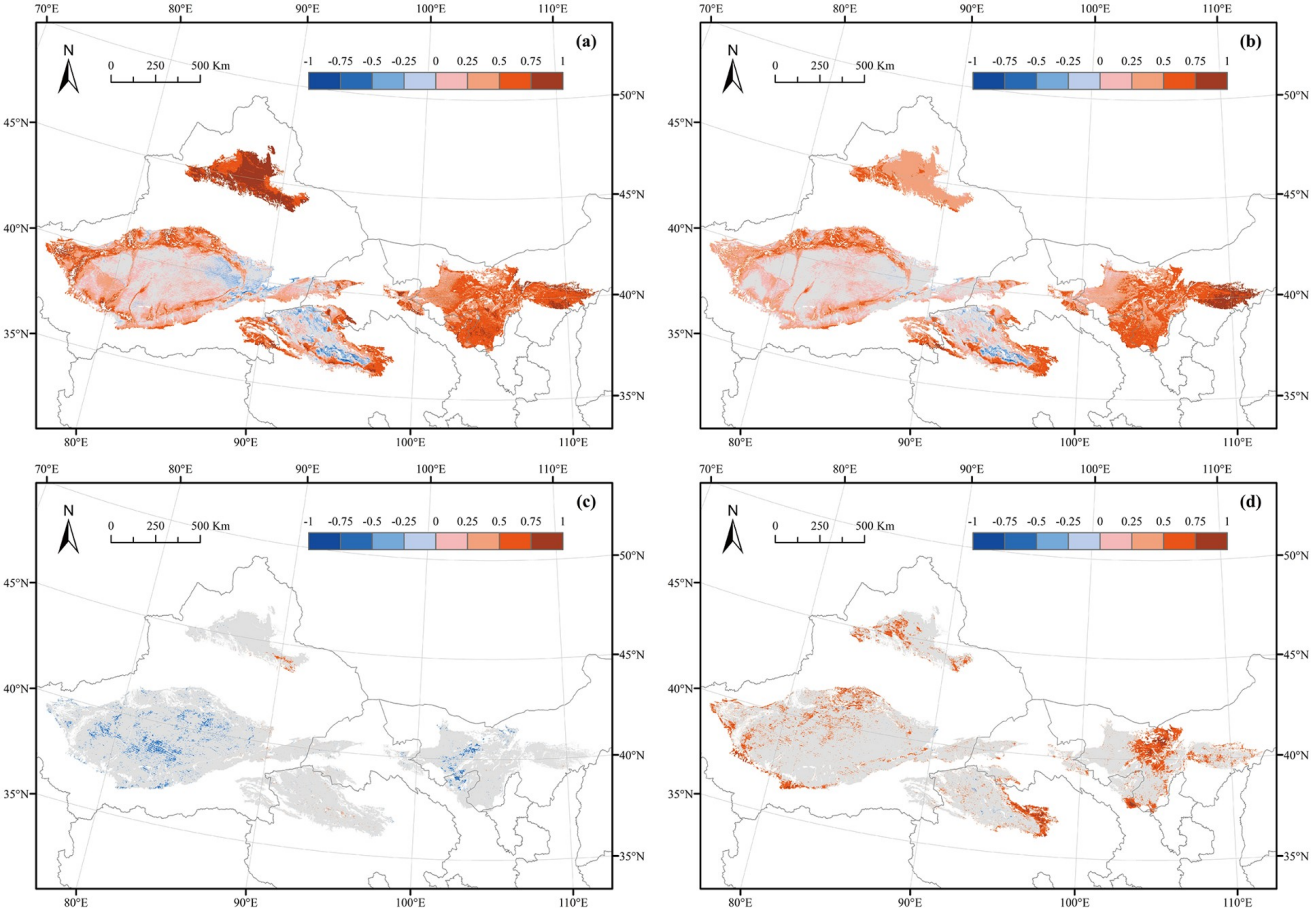
Spatial distribution of the Pearson correlation coefficient for the relationships between the NDVI and climatic factors. (a) Correlation between the monthly NDVI and temperature. (b) Correlation between the monthly NDVI and precipitation. (c) Correlation between the annual NDVI and temperature. (d) Correlation between the annual NDVI and precipitation.

Fig 6(a) illustrates the spatial distribution of the temperature effect on the NDVI at the monthly scale; 94.15% of the area in which the NDVI had a positive correlation with temperature was distributed in areas other than the eastern part of the Taklimakan Desert and the central part of the Qaidam Basin Desert. The strong positive correlation was principally distributed in the Gurbantunggut Desert, the western edge of the Taklimakan Desert, the western and eastern edges of the Qaidam Basin Desert, the Tengger Desert, and the Kubuqi Desert, indicating the area where the NDVI showed significant greening trends.

Fig 6(b) illustrates the spatial distribution of the influence of precipitation on NDVI at the monthly scale, which was similar to that of temperature; however, the correlation in the Gurbantunggut Desert was not as strong as that of temperature. Overall, 83.28% of the area showed a positive correlation, and 68.19% was significant (p<0.05), which was lower than that of temperature. In addition, 16.72% of the area had a negative correlation; this area was distributed in the eastern part of the Taklimakan Desert and the central part of the Qaidam Basin Desert.

The correlations between the annual NDVI and climatic factors were not significant in the majority of pixels, which were much weaker than the correlations between the climatic factors and the monthly NDVI. In the very few pixels with significant correlation, temperature mainly had a negative effect on the NDVI, while precipitation had a positive effect and had a larger area than temperature. Specifically, the percentages of the significant positive and negative correlations with temperature were only 0.50% and 3.98%, respectively, and those with precipitation were only 14.99% and 0.15%, respectively. In terms of spatial distribution, the positive correlation with temperature was mostly in the southeastern part of the Gurbantunggut Desert, the Qaidam Basin Desert, and the Ulan Buh Desert, and the negative correlation was mostly in the western part of the Gurbantunggut Desert, the midwestern part of the Taklimakan Desert, and the Badain Jaran Desert. In contrast, the positive correlation with precipitation was distributed in most areas, with a slight negative correlation in the mideastern part of the Taklimakan Desert and the central part of the Qaidam Basin Desert.

### Impact of human activities

**Fig 7:**
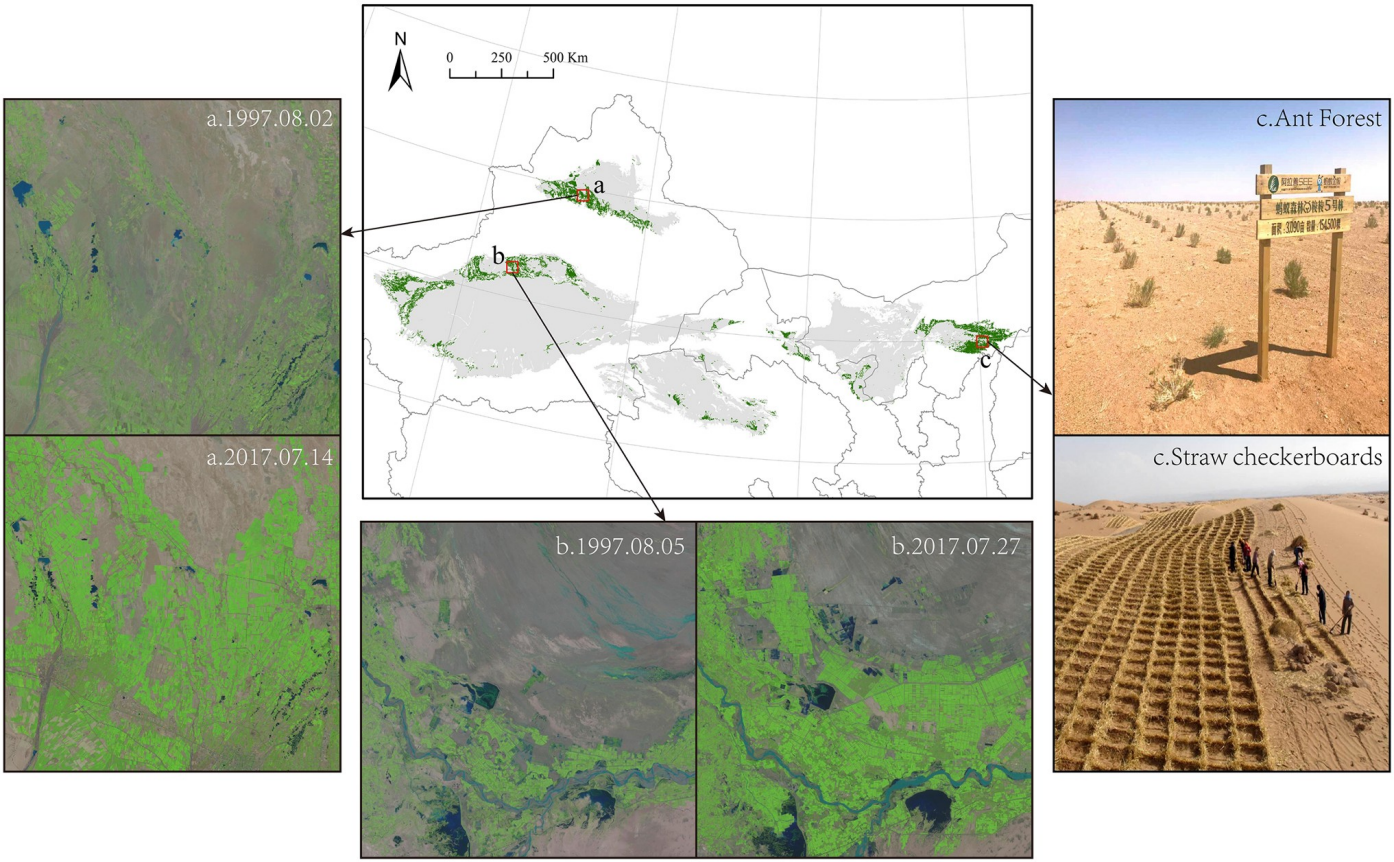
Greening regions caused by human activities. Green areas denote desert regions where greening trends were influenced by human activities.

The impact of human activities was calculated by residual analysis. Areas where human activities caused NDVI variations were greater than climate variability were classified as human activities impact areas. Fig 7 shows the region where NDVI variations were caused by human activities, where considerable greening trends were observed. This region included the southern part of the Gurbantunggut Desert, the edge of the Taklimakan Desert, and the Kubuqi Desert.

To further identify which human activities caused the deserts greening, we compared Landsat images and Google Earth images of the desert greening areas with those of the desert non-greening areas. The images for 2000 and 2017 indicate that land cover changes can be clearly observed. By comparison, there were two main types of human activities that caused deserts greening: oasis expansion and sand stabilization measures. Specifically, the areas showed greening trends in the southern part of the Gurbantunggut Desert, the northwestern edge of the Taklimakan Desert, the eastern Kumtag Desert, the Badain Jaran Desert, and the Tengger Desert were transformed into cropland due to the expansion of oasis. The greening areas in the Kubuqi Desert changed into shrubs and grass grids due to sand stabilization measures. Due to the space limitation, only a few image comparisons before and after the greening of the deserts caused by human activities are shown in Fig 7. More remote sensing image evidence about oasis expansion and sand stabilization measures as the two major factors of desert greening in China’s deserts are listed in the Supplementary Materials.

## Discussion

### Variations in the NDVI and its relationship with the climatic factors

China’s deserts showed significant greening trends both temporally and spatially, reflecting the increased vegetation cover of China’s deserts during the study period. Desert greening has far-reaching impacts on climate variability and environmental improvement. Firstly, it increases the land space available for human use, and the increased vegetation cover in deserts increases the carbon sink capacity. Secondly, increased vegetation helps improve the water cycle and increases local air humidity, thus improving the global climate [69,70]. In addition, it also reduces dust storm weather in northern China and improves air quality [71,72].

In a similar period, greening was observed not only in the China’s deserts, but also in other arid and semiarid regions [19]. For example, a slight increase was observed in more than 80% of the arid mountain-oasis river basin in northwestern China [73]. In the Hexi region, approximately 70% of the vegetation area presented a significant increasing trend, and only 2.85% presented a significant decreasing trend [74,75]. And a significant increasing trend of the NDVI was observed in Xinjiang [76]. In general, precipitation has been shown to be the main cause of NDVI rise in arid and semiarid regions, such as the Hexi region, Xinjiang, and Central Asia [34,73,75,76]. However, from this study, although the correlation between precipitation and NDVI was significant on the annual scale, the area with significant correlation was only 15.14%. The positive effect of precipitation and the negative effect of temperature on NDVI, and the greater effect of precipitation compared to temperature were also observed in related studies [34,36,56,77,78], mainly because the vegetation growth in arid regions is more limited due to the lack of moisture.

The lagged effects of climatic factors on NDVI were not observed at the monthly scale, which may be because the time scale was not fine enough that the lagged effect might be less than one month. Related studies show that in temperate grassland deserts, the average lag time between NDVI and temperature and precipitation is 16.4 days and 11.8 days, respectively [79].

In general, the correlations at the seasonal and monthly scales were significantly strong but weaker at the annual scale. Spatially, the correlations with the NDVI at the monthly scale were stronger, and those at the annual scale were weaker and showed a completely different spatial distribution. These results confirm that climate variability is the main explanation of intra-annual NDVI variations, but could not effectively explain the greening and browning trends of China’s deserts from 2000 to 2017 reflected by NDVI at the annual scale.

### Human activities

The results show that the majority of the desert area showed a significantly increasing trend from 2000 to 2017. By comparing remote sensing images of all desert areas that had turned green with those that had not, we found that there are two principal human activities contributing to the greening of deserts in China: oasis expansion and sand stabilization measures (Fig 7). These two anthropogenic impacted desert areas differ. Oasis expansion, especially in the form of cropland expansion, led to considerable greening areas that were mainly distributed in the southern part of the Gurbantunggut Desert, the northwestern edge of the Taklimakan Desert, patches at the edges of the Qaidam Basin Desert, the Badain Jaran Desert, and the Tengger Desert. The expansion of crops in the desert region can be connected to inadequate transfer of rural labor forces and grain subsidies, which can lead to excessive consumption of water and disrupt the natural balance between the oasis and the desert [80]. Policies to control the moderate expansion of oasis need to be strengthened like facilitating the transfer of agricultural labor to non-agricultural labor and reasonably controlling the grain subsidies in these areas to effectively control the expansion of oasis. Similarly, agricultural practices are the primary cause of greening trends in most regions of the world [22,36]. Sand stabilization measures such as using straw checkerboards and planting grass, shrubs, and trees contributed to quite a few greening areas in the Kubuqi Desert. This result provides evidence of the effectiveness of desertification control in China [81]. By employing sustainable grazing practices, sustainable agriculture practices, water use quotas, and planting wind-shelter forests, China has paid great attention to desertification management [82]. These efforts in China provide a valuable example for sustainable desertified land management throughout the world [83]. Since 2000, desertification in China has been implemented through national projects (e.g., the Sandstorm Source Control project and the Three-North Shelterbelt System Construction) [82,84]. Despite great progress in the greening of desert areas in China, there is still a long way to go, indicating the need for increased efforts by the government and funding for effective policies and projects [85].

## Conclusions

This study analyzed greening and browning trends in China’s deserts from 2000 to 2017 shown in the monthly, seasonal, and annual NDVI both temporally and spatially and measured the effects of climate variability and human activities by correlation analysis and residual analysis. The following conclusions were obtained.

The annual NDVI showed a significant increasing trend at a rate of 0.0018/yr, with values of 0.094 in 2000 and 0.126 in 2017. At the seasonal scale, the mean values of the NDVI were higher in summer and autumn, and lower in spring and winter, which showed significant increases in all four seasons, also higher in summer and autumn, and lower in spring and winter. The monthly NDVI showed an inverted U-shaped curve during the year, which was the lowest in January (0.0539) and the highest in August (0.0989). The variation of the monthly NDVI trends was similar. In terms of the climatic factors, the variations in monthly, seasonal, and annual temperature and precipitation did not show significant trends from 2000 to 2017.

A total of 62.8% of the desert area showed greening trends, and only 0.78% showed browning trends in spatial. The greening trends were mainly distributed on the southern edge of the Gurbantunggut Desert, in the northwestern part of the Taklimakan Desert, and in the Kubuqi Desert. The browning trends were distributed in the northwestern part of the Taklimakan Desert and the eastern part of the Qaidam Basin Desert. In terms of longitude and latitude, the area with greening trends was positively correlated with the desert area. The Badain Jaran Desert showed the highest percentage of greening (88.26%), followed by the Kumtag Desert (75.26%) and the Tengger Desert (70.07%); however, the Qaidam Basin Desert showed the highest percentage of browning (1.25%). In terms of seasons, spring had the largest proportion of greening area, followed by summer, winter, and autumn. The spatial distribution of the greening and browning trends showed seasonal differences, and the spatial pattern in summer was the most similar to that at the annual scale, followed by autumn, winter, and spring.

At the annual scale, climatic factors had limited explanatory power for NDVI variations, and only precipitation had a significant effect on NDVI, and the effect of precipitation was stronger than that of temperature. At the seasonal and monthly scales, temperature and precipitation were the main factors affecting NDVI variations. Furthermore, the lagged effects of climate factors on NDVI at monthly scales were not observed because the time scale was not fine enough.

Human activities played a critical role in the greening of China’s deserts from 2000 to 2017. Specifically, oasis expansion and sand stabilization measures were the two major reasons that led to desert greening. The former occurred mainly in the southern part of the Gurbantunggut Desert, the northwestern edge of the Taklimakan Desert, and patches at the edges of the Qaidam Basin Desert, the Badain Jaran Desert, and the Tengger Desert, while the latter occurred mainly in the Kubuqi Desert.

## Supporting information

S1 File Image evidence of human activities. (PDF)

S2 File Data set (part 1). (ZIP)

S3 File Data set (part 2). (ZIP)

S4 File Data set (part 3). (ZIP)

S5 File Data set (part 4). (ZIP)

S6 File Data set (part 5). (ZIP)

S7 File Data set (part 6). (ZIP)

S8 File Data set (part 7). (ZIP)

## References

[1] Mabbutt JA. Desert landforms: Australian National University Press; 1977.

[2] Cooke RU, Warren A, Goudie AS. Desert geomorphology: CRC Press; 1993.

[3] Whitford WG, Duval BD. Ecology of desert systems: Academic Press; 2019.

[4] Warner TT. Desert meteorology: Cambridge University Press; 2009.

[5] Laity JJ. Deserts and desert environments: John Wiley & Sons; 2009.

[6] Stocker TF, Qin D, Plattner G-K, Tignor M, Allen SK, Boschung J, et al. Climate change 2013: The physical science basis. Contribution of working group I to the fifth assessment report of the intergovernmental panel on climate change. 2013;1535.

[7] Kim J, Hogue TS. Evaluation and sensitivity testing of a coupled Landsat-MODIS downscaling method for land surface temperature and vegetation indices in semi-arid regions. Journal of Applied Remote Sensing. 2012;6(1):063569.

[8] Xu Y, Yang J, Chen Y. NDVI-based vegetation responses to climate change in an arid area of China. Theoretical and Applied Climatology. 2016;126(1–2):213–22.

[9] Zhou J, Cai W, Qin Y, Lai L, Guan T, Zhang X, et al. Alpine vegetation phenology dynamic over 16 years and its covariation with climate in a semi-arid region of China. Science of the Total Environment. 2016;572:119–28. 10.1016/j.scitotenv.2016.07.206 27494658

[10] Yin H, Li Z, Wang Y, Cai F. Assessment of desertification using time series analysis of hyper-temporal vegetation indicator in Inner Mongolia. Acta Geographica Sinica. 2011;66(5):653–61.

[11] Leprieur C, Kerr Y, Mastorchio S, Meunier J. Monitoring vegetation cover across semi-arid regions: comparison of remote observations from various scales. International Journal of Remote Sensing. 2000;21(2):281–300.

[12] Goward SN, Tucker CJ, Dye DG. North American vegetation patterns observed with the NOAA-7 advanced very high resolution radiometer. Vegetatio. 1985;64(1):3–14.

[13] Malingreau J, Tucker C, Laporte N. AVHRR for monitoring global tropical deforestation. International Journal of Remote Sensing. 1989;10(4–5):855–67.

[14] Martiny N, Camberlin P, Richard Y, Philippon N. Compared regimes of NDVI and rainfall in semi‐arid regions of Africa. International Journal of Remote Sensing. 2006;27(23):5201–23.

[15] Olusegun CF, Adeyewa ZD. Spatial and temporal variation of normalized difference vegetation index (NDVI) and rainfall in the north east arid zone of Nigeria. Atmospheric and Climate Sciences. 2013;2013.

[16] Weiss JL, Gutzler DS, Coonrod JEA, Dahm CN. Long-term vegetation monitoring with NDVI in a diverse semi-arid setting, central New Mexico, USA. Journal of Arid Environments. 2004;58(2):249–72.

[17] Li Y, Liu Y, Zhang P, YIN Y-h. Research on the spatio-temporal change of NDVI in the Gurbantunggut Desert. Arid Zone Research. 2009;26(5):686–93.

[18] Propastin P, Kappas M, Muratova N. Inter-Annual Changes in Vegetation Activities and Their Relationship to Temperature and Precipitation in Central Asia from 1982 to 2003. Journal of Environmental Informatics. 2008;12(2).

[19] Fensholt R, Langanke T, Rasmussen K, Reenberg A, Prince SD, Tucker C, et al. Greenness in semi-arid areas across the globe 1981–2007—an Earth Observing Satellite based analysis of trends and drivers. Remote sensing of environment. 2012;121:144–58.

[20] Kawabata A, Ichii K, Yamaguchi Y. Global monitoring of interannual changes in vegetation activities using NDVI and its relationships to temperature and precipitation. International journal of remote sensing. 2001;22(7):1377–82.

[21] Formica AF, Burnside RJ, Dolman PM. Rainfall validates MODIS-derived NDVI as an index of spatio-temporal variation in green biomass across non-montane semi-arid and arid Central Asia. Journal of Arid Environments. 2017;142:11–21.

[22] Chen C, Park T, Wang X, Piao S, Xu B, Chaturvedi RK, et al. China and India lead in greening of the world through land-use management. Nature sustainability. 2019;2(2):122–9. doi: 10.1038/s41893-019-0220-7 30778399 PMC6376198

[23] Evans J, Geerken R. Discrimination between climate and human-induced dryland degradation. Journal of arid environments. 2004;57(4):535–54.

[24] Wessels KJ, Prince S, Malherbe J, Small J, Frost P, VanZyl D. Can human-induced land degradation be distinguished from the effects of rainfall variability? A case study in South Africa. Journal of arid environments. 2007;68(2):271–97.

[25] He B, Chen A, Wang H, Wang Q. Dynamic response of satellite-derived vegetation growth to climate change in the Three North Shelter Forest Region in China. Remote Sensing. 2015;7(8):9998–10016.

[26] Ichii K, Kawabata A, Yamaguchi Y. Global correlation analysis for NDVI and climatic variables and NDVI trends: 1982–1990. International journal of remote sensing. 2002;23(18):3873–8.

[27] Zhou L, Tucker CJ, Kaufmann RK, Slayback D, Shabanov NV, Myneni RB. Variations in northern vegetation activity inferred from satellite data of vegetation index during 1981 to 1999. Journal of Geophysical Research: Atmospheres. 2001;106(D17):20069–83.

[28] Karnieli A, Agam N, Pinker RT, Anderson M, Imhoff ML, Gutman GG, et al. Use of NDVI and land surface temperature for drought assessment: Merits and limitations. Journal of climate. 2010;23(3):618–33.

[29] Wang J, Rich PM, Price KP. Temporal responses of NDVI to precipitation and temperature in the central Great Plains, USA. International journal of remote sensing. 2003;24(11):2345–64.

[30] Liu Y, Li Y, Li S, Motesharrei S. Spatial and temporal patterns of global NDVI trends: correlations with climate and human factors. Remote Sensing. 2015;7(10):13233–50.

[31] Miao L, Jiang C, Xue B, Liu Q, He B, Nath R, et al. Vegetation dynamics and factor analysis in arid and semi-arid Inner Mongolia. Environmental Earth Sciences. 2015;73(5):2343–52.

[32] Noy-Meir I. Desert ecosystems: environment and producers. Annual review of ecology and systematics. 1973;4(1):25–51.

[33] Mohammat A, Wang X, Xu X, Peng L, Yang Y, Zhang X, et al. Drought and spring cooling induced recent decrease in vegetation growth in Inner Asia. Agricultural and Forest Meteorology. 2013;178:21–30.

[34] Ren J, Liu H, Yin Y, He S. Drivers of greening trend across vertically distributed biomes in temperate arid Asia. Geophysical research letters. 2007;34(7).

[35] Mao D, Wang Z, Luo L, Ren C. Integrating AVHRR and MODIS data to monitor NDVI changes and their relationships with climatic parameters in Northeast China. International Journal of Applied Earth Observation and Geoinformation. 2012;18:528–36.

[36] Piao S, Fang J, Zhou L, Guo Q, Henderson M, Ji W, et al. Interannual variations of monthly and seasonal normalized difference vegetation index (NDVI) in China from 1982 to 1999. Journal of Geophysical Research: Atmospheres. 2003;108(D14).

[37] Braswell B, Schimel DS, Linder E, Moore B. The response of global terrestrial ecosystems to interannual temperature variability. Science. 1997;278(5339):870–3.

[38] Szabó S, Elemér L, Kovács Z, Püspöki Z, Kertész Á, Singh SK, et al. NDVI dynamics as reflected in climatic variables: spatial and temporal trends–a case study of Hungary. GIScience & Remote Sensing. 2019;56(4):624–44.

[39] Jiang R, Xie J, He H, Kuo C-C, Zhu J, Yang M. Spatiotemporal variability and predictability of normalized difference vegetation index (NDVI) in Alberta, Canada. International journal of biometeorology. 2016;60(9):1389–403. doi: 10.1007/s00484-015-1132-5 26768143

[40] Paruelo JM, Lauenroth WK. Interannual variability of NDVI and its relationship to climate for North American shrublands and grasslands. Journal of Biogeography. 1998;25(4):721–33.

[41] Duan H, Yan C, Tsunekawa A, Song X, Li S, Xie J. Assessing vegetation dynamics in the Three-North Shelter Forest region of China using AVHRR NDVI data. Environmental Earth Sciences. 2011;64(4):1011–20.

[42] Victoria DdC, Paz ARd, Coutinho AC, Kastens J, Brown JC. Cropland area estimates using Modis NDVI time series in the state of Mato Grosso, Brazil. Pesquisa Agropecuária Brasileira. 2012;47(9):1270–8.

[43] Nanzad L, Zhang J, Tuvdendorj B, Nabil M, Zhang S, Bai Y. NDVI anomaly for drought monitoring and its correlation with climate factors over Mongolia from 2000 to 2016. Journal of arid environments. 2019;164:69–77.

[44] Wang J, Wang Y, Yan C, Qi Y. 1:100,000 desert (sand) distribution dataset in China. In: Center NTPD, editor. 2013.

[45] Wang J. The map of desert distribution in 1:2,000,000 in China (1974). In: Center NTPD, editor. 2013.

[46] Didan K. MOD13A2 MODIS/Terra Vegetation indices 16-Day L3 Global 1km SIN Grid V006 [Data set]. NASA EOSDIS LP DAAC https://doiorg/105067/MODIS/MOD13A22015.

[47] Holben BN. Characteristics of maximum-value composite images from temporal AVHRR data. International journal of remote sensing. 1986;7(11):1417–34.

[48] Li H, Li Y, Gao Y, Zou C, Yan S, Gao J. Human impact on vegetation dynamics around Lhasa, southern Tibetan plateau, China. Sustainability. 2016;8(11):1146.

[49] Stow DA, Hope A, McGuire D, Verbyla D, Gamon J, Huemmrich F, et al. Remote sensing of vegetation and land-cover change in Arctic Tundra Ecosystems. Remote sensing of environment. 2004;89(3):281–308.

[50] Ding Y, Peng S. Spatiotemporal Trends and Attribution of Drought across China from 1901–2100. Sustainability. 2020;12(2):477.

[51] Peng S, Ding Y, Liu W, Li Z. 1 km monthly temperature and precipitation dataset for China from 1901 to 2017. Earth System Science Data. 2019;11(4).

[52] Peng S, Ding Y, Wen Z, Chen Y, Cao Y, Ren J. Spatiotemporal change and trend analysis of potential evapotranspiration over the Loess Plateau of China during 2011–2100. Agricultural and forest meteorology. 2017;233:183–94.

[53] Fernandes R, Leblanc SG. Parametric (modified least squares) and non-parametric (Theil–Sen) linear regressions for predicting biophysical parameters in the presence of measurement errors. Remote Sensing of Environment. 2005;95(3):303–16.

[54] Sen PK. Estimates of the regression coefficient based on Kendall’s tau. Journal of the American statistical association. 1968;63(324):1379–89.

[55] Arrogante-Funes P, Novillo CJ, Romero-Calcerrada R. Monitoring NDVI Inter-Annual Behavior in Mountain Areas of Mainland Spain (2001–2016). Sustainability. 2018;10(12):4363.

[56] Baniya B, Tang Q, Huang Z, Sun S, Techato K-a. Spatial and temporal variation of NDVI in response to climate change and the implication for carbon dynamics in Nepal. Forests. 2018;9(6):329.

[57] Gillespie TW, Ostermann-Kelm S, Dong C, Willis KS, Okin GS, MacDonald GM. Monitoring changes of NDVI in protected areas of southern California. Ecological Indicators. 2018;88:485–94.

[58] Yang Y, Wang S, Bai X, Tan Q, Li Q, Wu L, et al. Factors affecting long-term trends in global NDVI. Forests. 2019;10(5):372.

[59] Kendall MG. Rank correlation methods. 1948.

[60] Mann HB. Nonparametric tests against trend. Econometrica: Journal of the econometric society. 1945:245–59.

[61] Fan D, Ni L, Jiang X, Fang S, Wu H, Zhang X. Spatiotemporal Analysis of Vegetation Changes Along the Belt and Road Initiative Region From 1982 to 2015. IEEE Access. 2020;8:122579–88.

[62] Herrmann SM, Anyamba A, Tucker CJ. Recent trends in vegetation dynamics in the African Sahel and their relationship to climate. Global Environmental Change. 2005;15(4):394–404.

[63] Zoungrana BJ, Conrad C, Thiel M, Amekudzi LK, Da ED. MODIS NDVI trends and fractional land cover change for improved assessments of vegetation degradation in Burkina Faso, West Africa. Journal of Arid Environments. 2018;153:66–75.

[64] Chu H, Venevsky S, Wu C, Wang M. NDVI-based vegetation dynamics and its response to climate changes at Amur-Heilongjiang River Basin from 1982 to 2015. Science of the Total Environment. 2019;650:2051–62. doi: 10.1016/j.scitotenv.2018.09.115 30290347

[65] Muradyan V, Tepanosyan G, Asmaryan S, Saghatelyan A, Dell’Acqua F. Relationships between NDVI and climatic factors in mountain ecosystems: A case study of Armenia. Remote Sensing Applications: Society and Environment. 2019;14:158–69.

[66] Luo H, Dai S, Xie Z, Fang J, editors. NDVI-Based analysis on the influence of human activities on vegetation variation on Hainan Island. IOP Conference Series: Earth and Environmental Science; IOP Publishing: Bristol, UK; 2018.

[67] Ma Q, Long Y, Jia X, Wang H, Li Y. Vegetation response to climatic variation and human activities on the Ordos Plateau from 2000 to 2016. Environmental Earth Sciences. 2019;78(24):709.

[68] Geerken R, Ilaiwi M. Assessment of rangeland degradation and development of a strategy for rehabilitation. Remote Sensing of Environment. 2004;90(4):490–504.

[69] Gao Y, Li X, Liu L, Jia R, Yang H, Li G, et al. Seasonal variation of carbon exchange from a revegetation area in a Chinese desert. Agricultural and forest meteorology. 2012;156:134–42.

[70] Li XR, Xiao HL, Zhang JG, Wang XP. Long‐term ecosystem effects of sand‐binding vegetation in the Tengger Desert, northern China. Restoration Ecology. 2004;12(3):376–90.

[71] An L, Che H, Xue M, Zhang T, Wang H, Wang Y, et al. Temporal and spatial variations in sand and dust storm events in East Asia from 2007 to 2016: Relationships with surface conditions and climate change. Science of The Total Environment. 2018;633:452–62. doi: 10.1016/j.scitotenv.2018.03.068 29579656

[72] Zou XK, Zhai PM. Relationship between vegetation coverage and spring dust storms over northern China. Journal of Geophysical Research: Atmospheres. 2004;109(D3).

[73] Mo K, Chen Q, Chen C, Zhang J, Wang L, Bao Z. Spatiotemporal variation of correlation between vegetation cover and precipitation in an arid mountain-oasis river basin in northwest China. Journal of Hydrology. 2019;574:138–47.

[74] Han J-C, Huang Y, Zhang H, Wu X. Characterization of elevation and land cover dependent trends of NDVI variations in the Hexi region, Northwest China. Journal of Environmental Management. 2019;232:1037–48. doi: 10.1016/j.jenvman.2018.11.069 33395756

[75] Yang X, Liu S, Yang T, Xu X, Kang C, Tang J, et al. Spatial-temporal dynamics of desert vegetation and its responses to climatic variations over the last three decades: a case study of Hexi region in Northwest China. Journal of Arid Land. 2016;8(4):556–68.

[76] Yu H, Bian Z, Mu S, Yuan J, Chen F. Effects of Climate Change on Land Cover Change and Vegetation Dynamics in Xinjiang, China. International Journal of Environmental Research and Public Health. 2020;17(13):4865. doi: 10.3390/ijerph17134865 32640654 PMC7370003

[77] Chuai X, Huang X, Wang W, Bao G. NDVI, temperature and precipitation changes and their relationships with different vegetation types during 1998–2007 in Inner Mongolia, China. International journal of climatology. 2013;33(7):1696–706.

[78] Piao S, Mohammat A, Fang J, Cai Q, Feng J. NDVI-based increase in growth of temperate grasslands and its responses to climate changes in China. Global Environmental Change-Human and Policy Dimensions. 2006;16(4):340–8. doi: 10.1016/j.gloenvcha.2006.02.002

[79] Kong D, Miao C, Wu J, Zheng H, Wu S. Time lag of vegetation growth on the Loess Plateau in response to climate factors: Estimation, distribution, and influence. Science of The Total Environment. 2020:140726. 10.1016/j.scitotenv.2020.140726 32693275

[80] Song W, Zhang Y. Expansion of agricultural oasis in the Heihe River Basin of China: Patterns, reasons and policy implications. Physics and Chemistry of the Earth, Parts A/B/C. 2015;89:46–55.

[81] Su YZ, Zhao WZ, Su PX, Zhang ZH, Wang T, Ram R. Ecological effects of desertification control and desertified land reclamation in an oasis–desert ecotone in an arid region: a case study in Hexi Corridor, northwest China. Ecological Engineering. 2007;29(2):117–24.

[82] Zhang Z, Huisingh D. Combating desertification in China: monitoring, control, management and revegetation. Journal of Cleaner Production. 2018;182:765–75.

[83] Lyu Y, Shi P, Han G, Liu L, Guo L, Hu X, et al. Desertification Control Practices in China. Sustainability. 2020;12(8):3258.

[84] Tao W. Aeolian desertification and its control in Northern China. International Soil and Water Conservation Research. 2014;2(4):34–41.

[85] Qi Y, Chang Q, Jia K, Liu M, Liu J, Chen T. Temporal-spatial variability of desertification in an agro-pastoral transitional zone of northern Shaanxi Province, China. Catena. 2012;88(1):37–45.

