# Supplementary material for: China’s deserts greening and response to climate variability and human activities
Source: PLoS One. 2021 Aug 30;16(8):e0256462. doi: 10.1371/journal.pone.0256462 (PMC8405022; doi:10.1371/journal.pone.0256462)

Supporting information for

China's deserts greening and response to climate variability and human activities

Xiaoyu Liu[1,2], Liangjie Xin[1,*]

[1] Key Laboratory of Land Surface Pattern and Simulation, Institute of Geographic Sciences and Natural Resources Research, Chinese Academy of Science, Beijing, China

[2] College of Resources and Environment, University of Chinese Academy of Sciences, Beijing, China

[*]Corresponding author.

E-mail: xinlj@igsnrr.ac.cn

The following shows the comparison of remote sensing images before and after the study period for the deserts greening areas dominated by human activities, reflecting that oasis expansion and sand stabilization measures are the two major anthropogenic factors for deserts greening in China.

1. Oasis expansion

The greening areas of deserts due to oasis expansion were mainly distributed in the southern part of the Gurbantunggut Desert, the northwestern edge of the Taklimakan Desert, the eastern Kumtag Desert, the Badain Jaran Desert, and the Tengger Desert (Number 1~57 in Fig S1). Images were from Landsat 4/5/8.

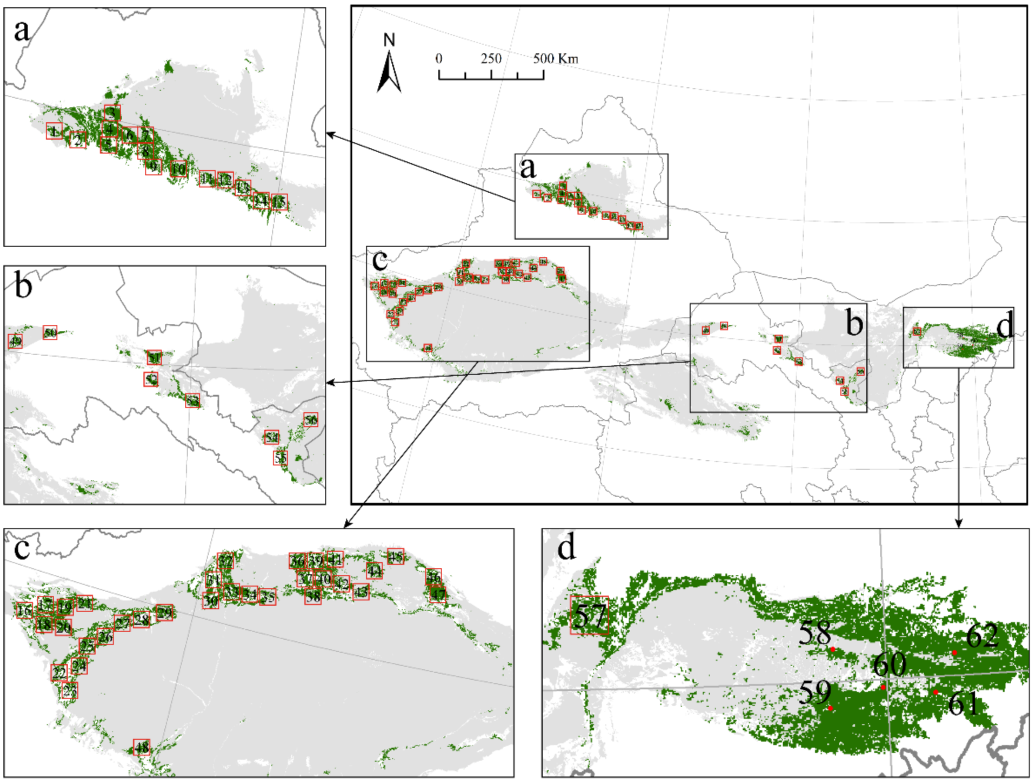

Fig S1 Locations of images evidence for greening areas of deserts dominated by human activities

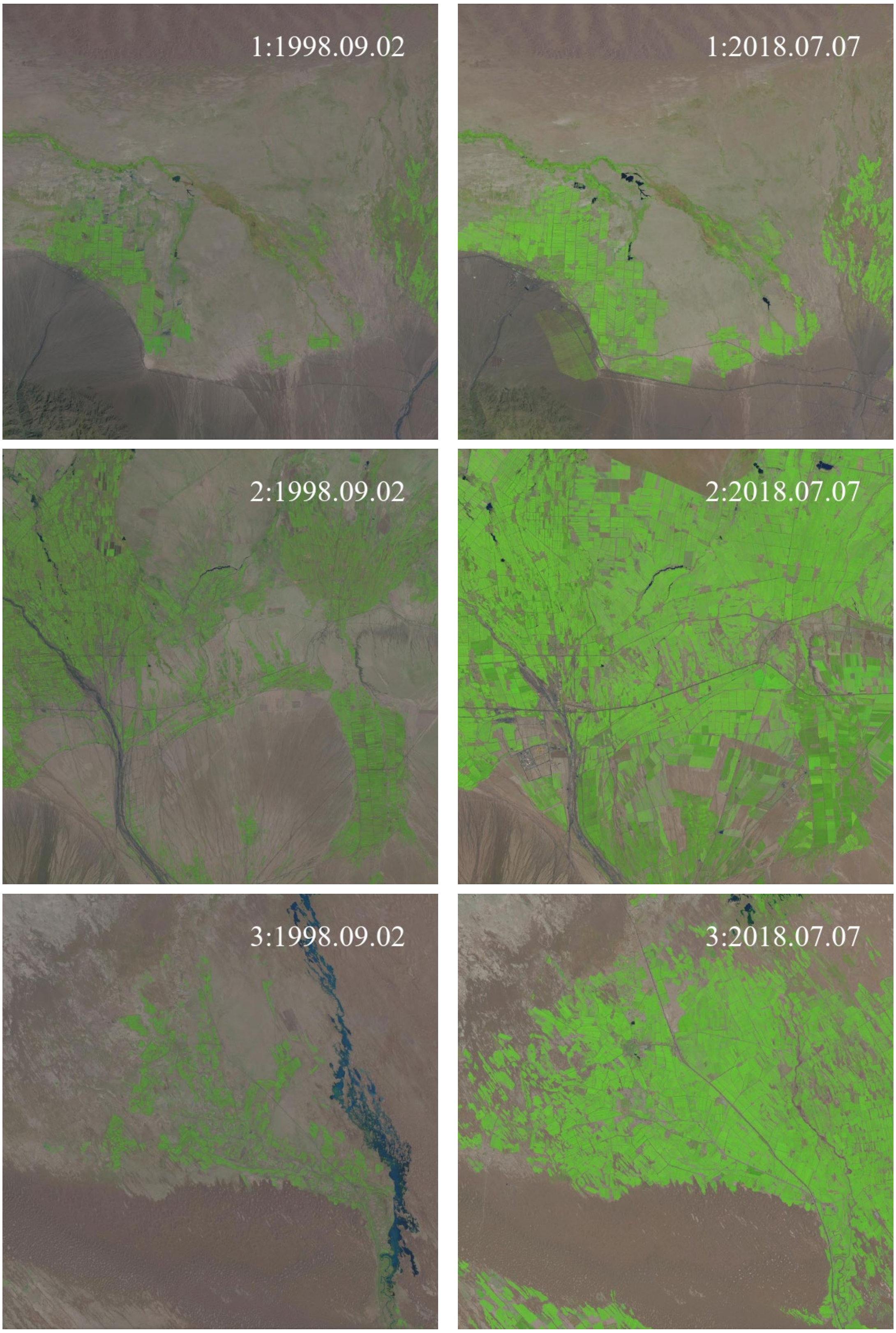
1:1998.09.02
1:2018.07.07
2:1998.09.02
2:2018.07.07
3:1998.09.02
3:2018.07.07

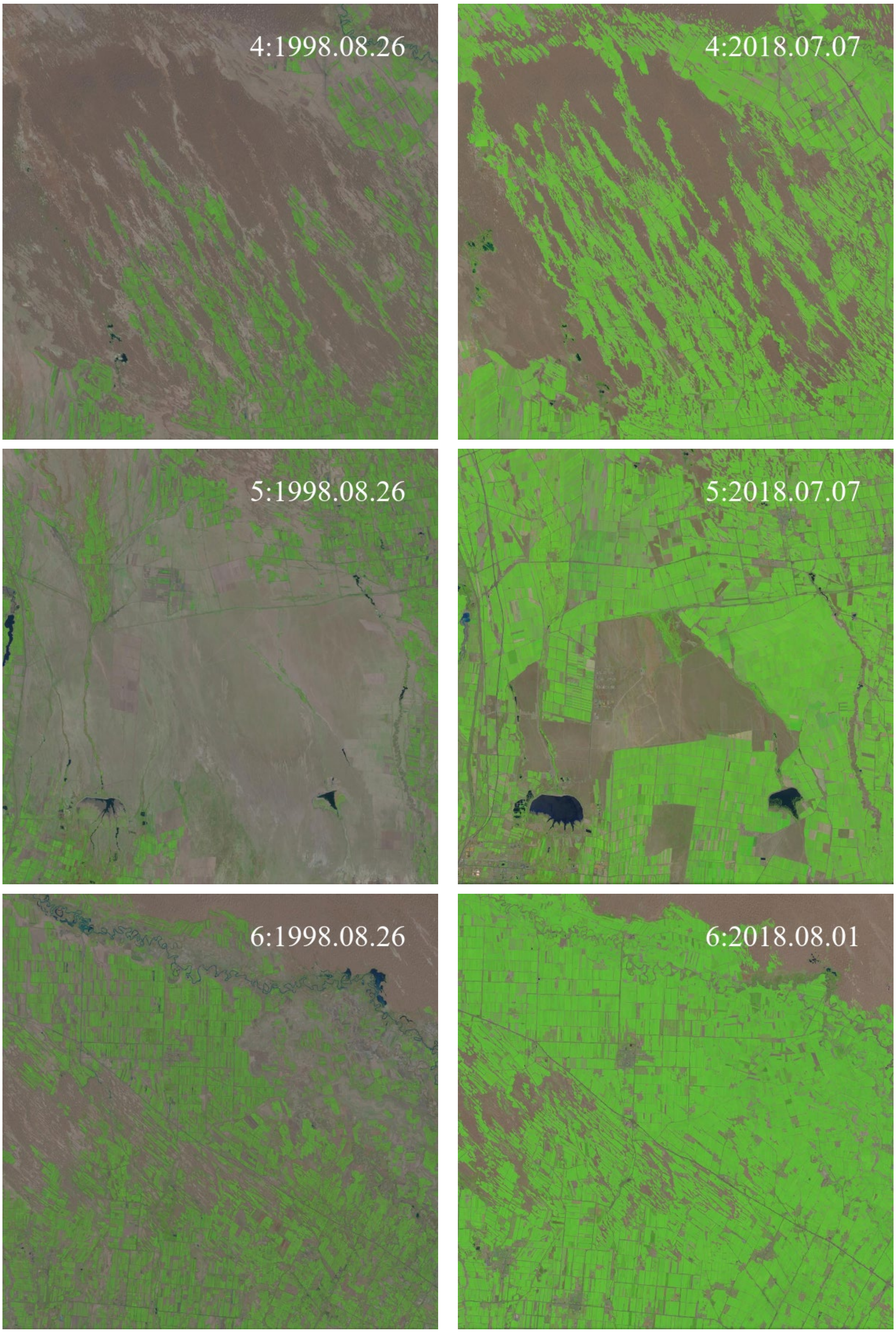
4:1998.08.26
4:2018.07.07
5:1998.08.26
5:2018.07.07
6:1998.08.26
6:2018.08.01

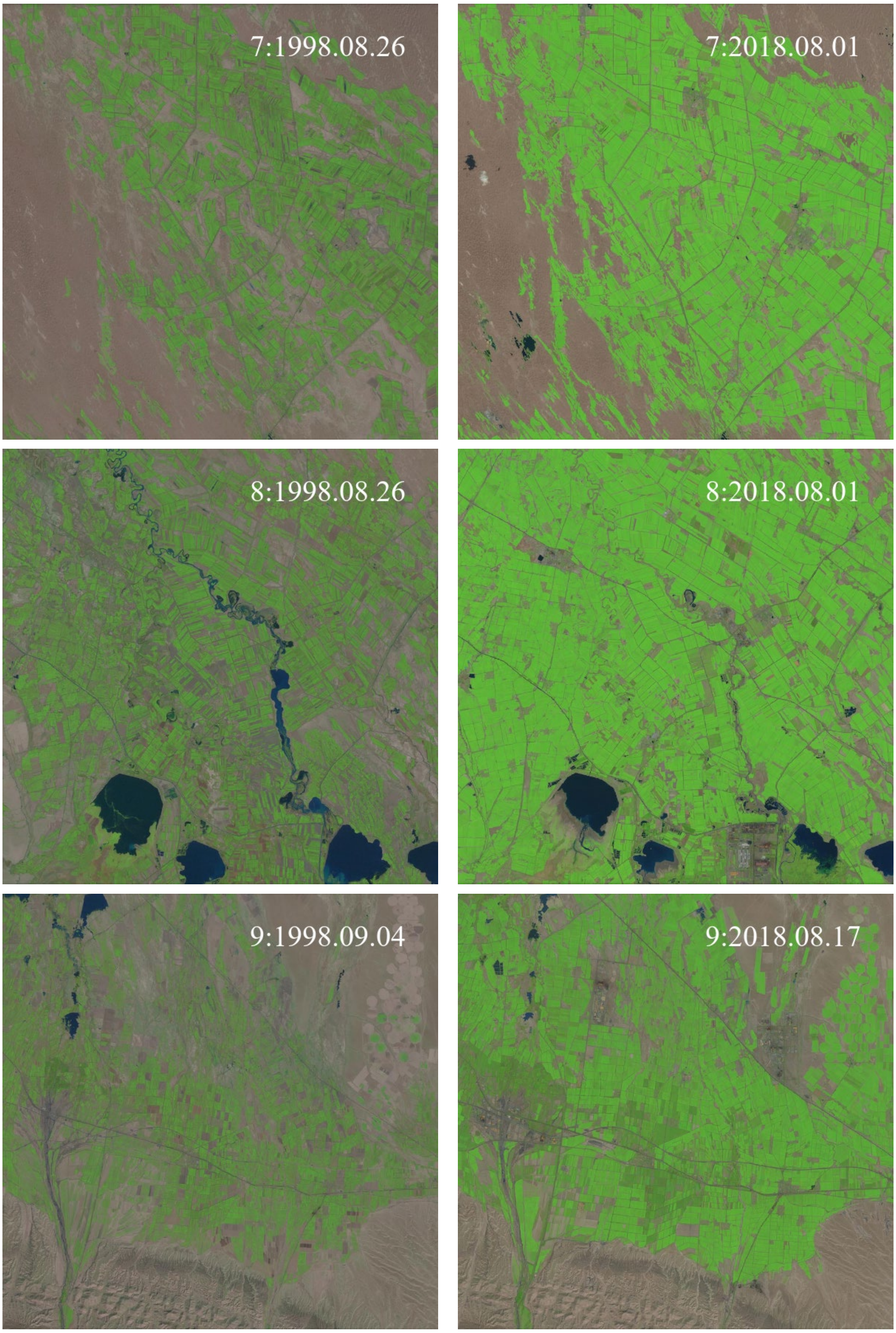
7:1998.08.26
7:2018.08.01
8:1998.08.26
8:2018.08.01
9:1998.09.04
9:2018.08.17

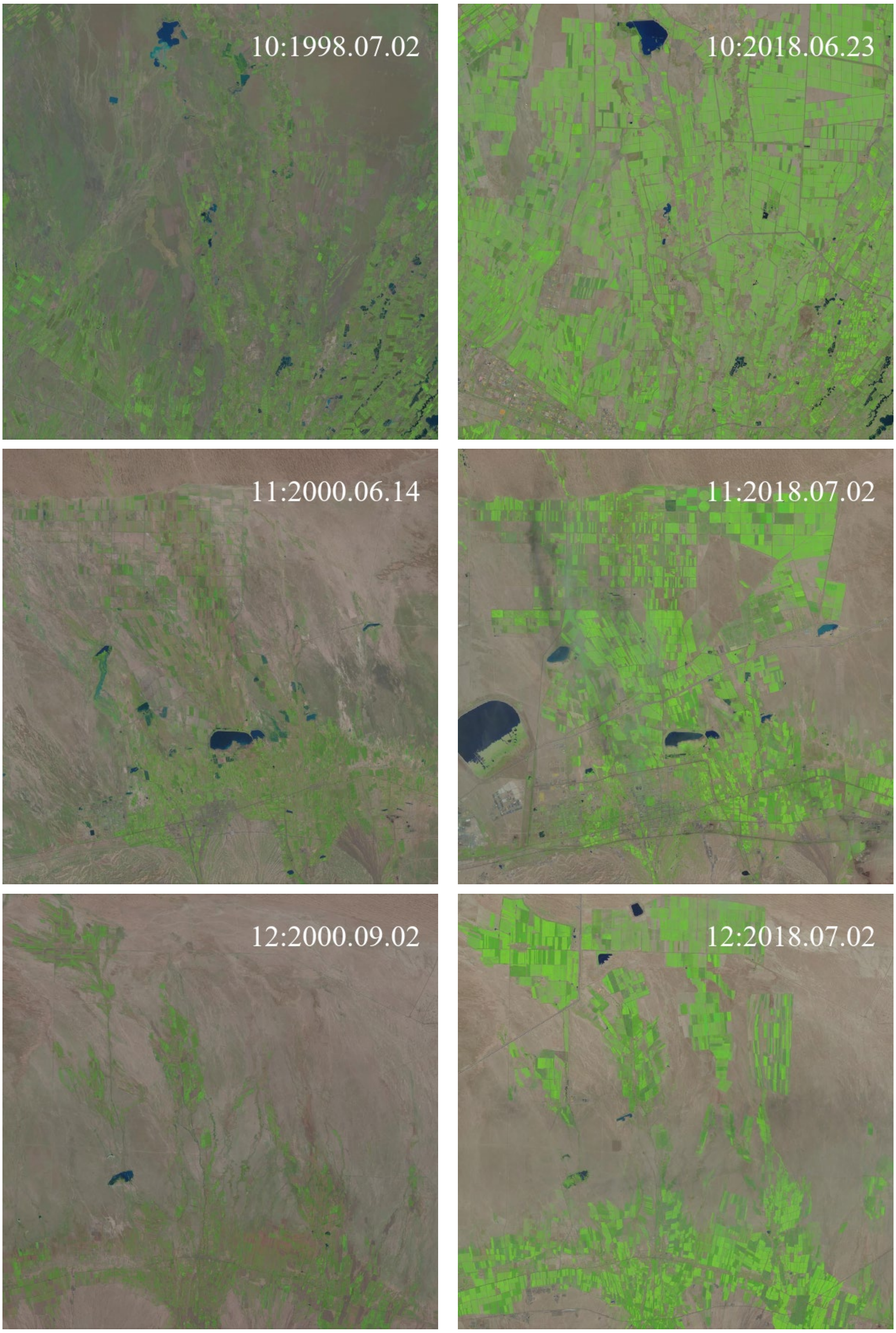
10:1998.07.02
10:2018.06.23
11:2000.06.14
11:2018.07.02
12:2000.09.02
12:2018.07.02

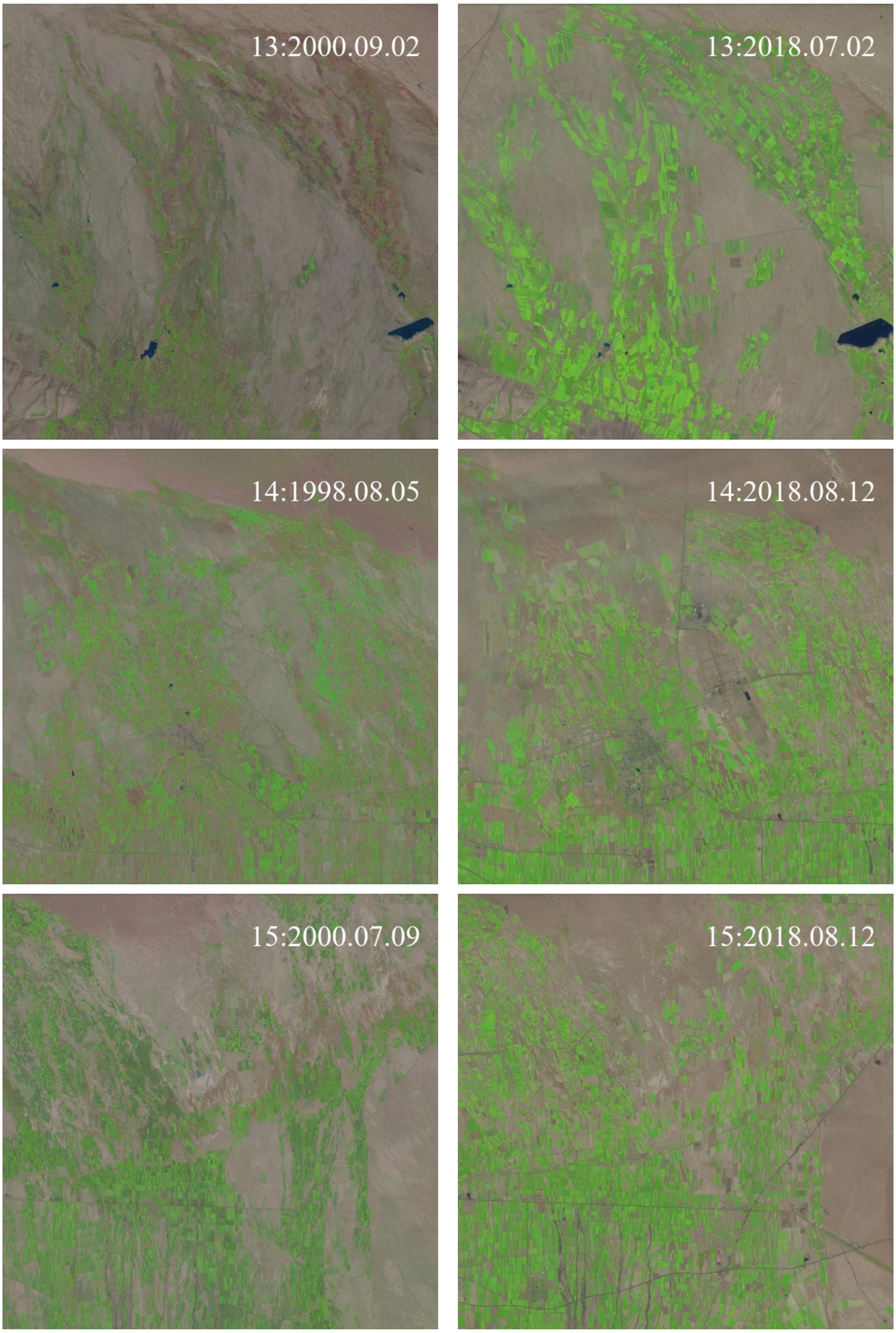

13:2000.09.02
13:2018.07.02
14:1998.08.05
14:2018.08.12
15:2000.07.09
15:2018.08.12

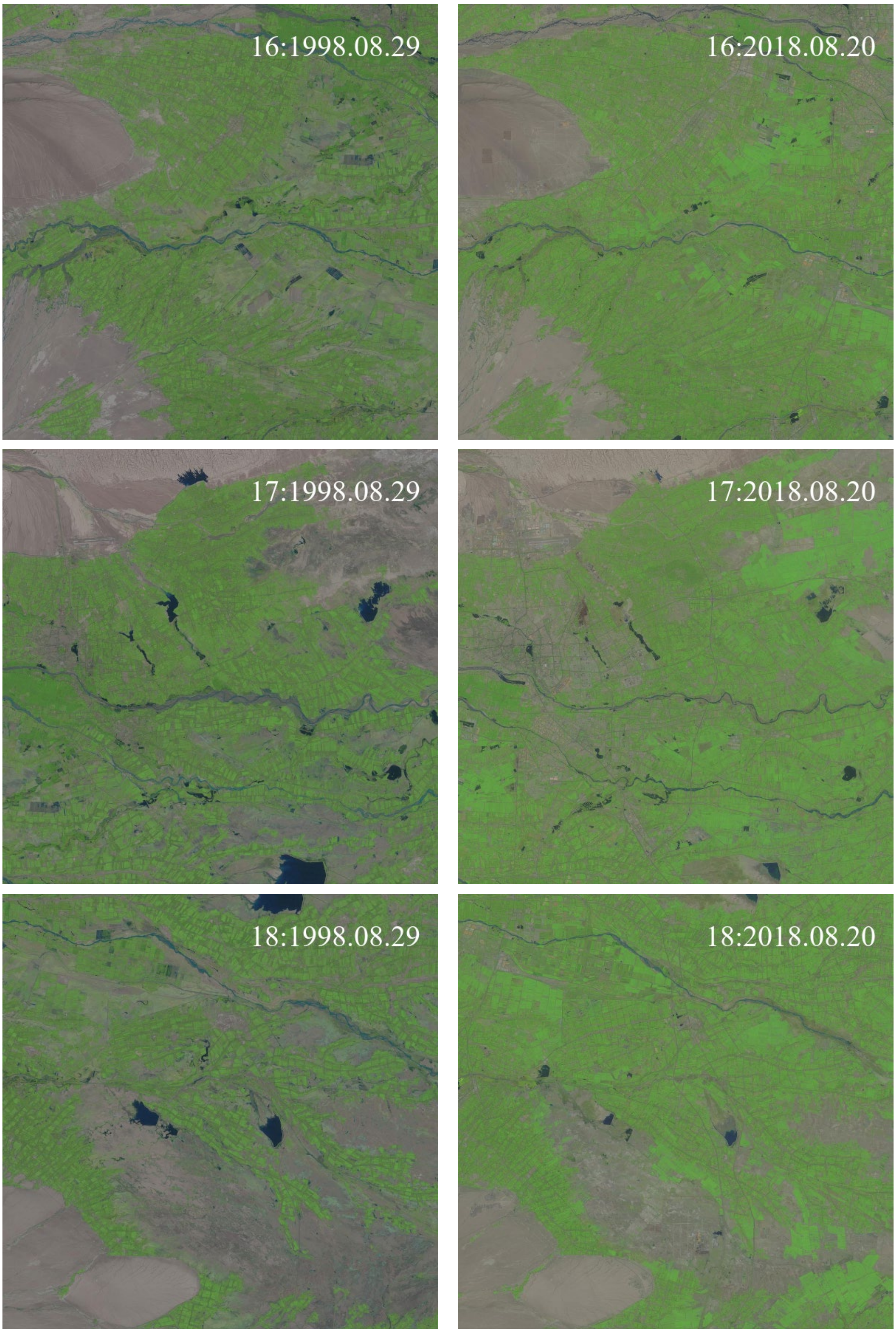
16:1998.08.29
16:2018.08.20
17:1998.08.29
17:2018.08.20
18:1998.08.29
18:2018.08.20

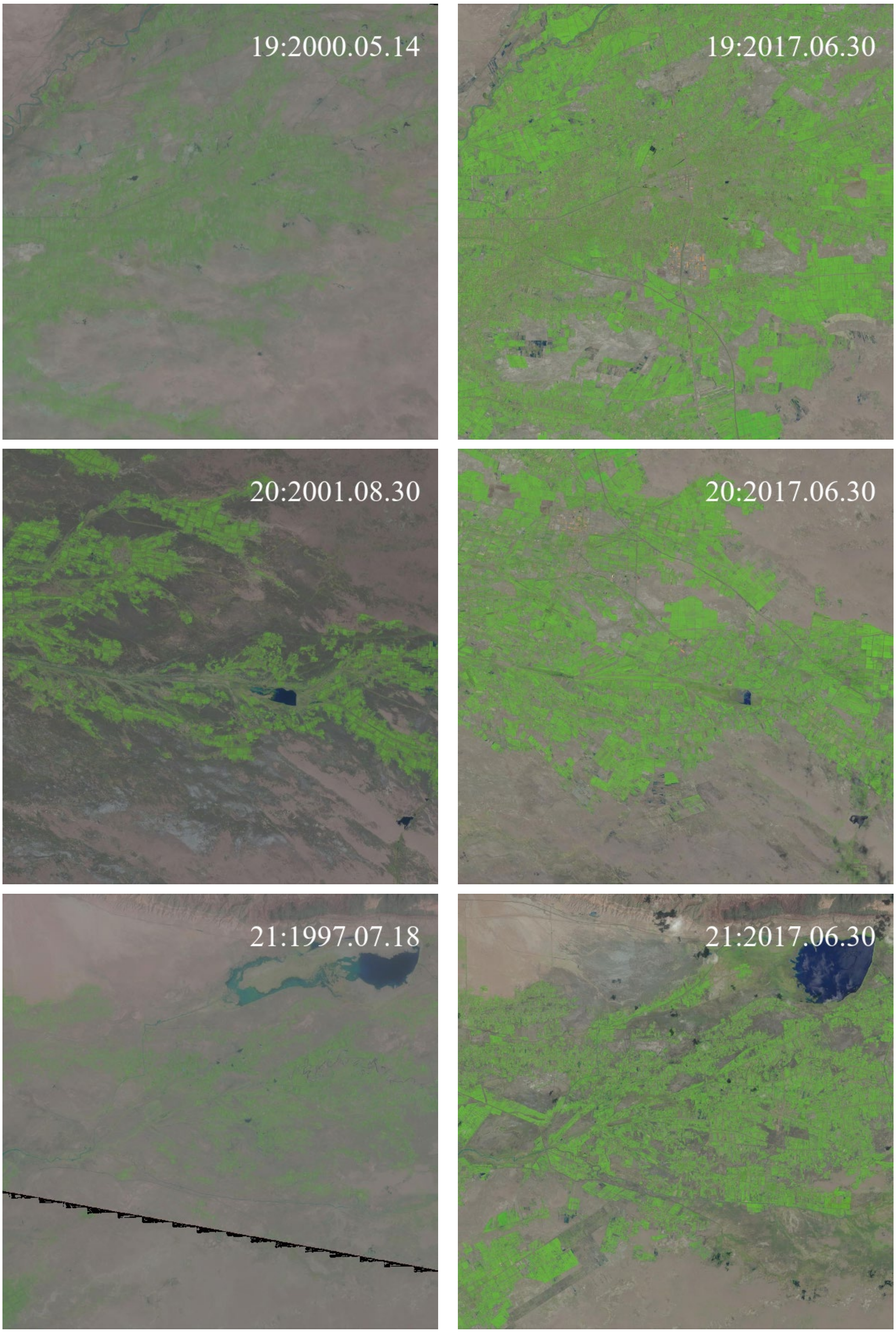
19:2000.05.14
19:2017.06.30
20:2001.08.30
20:2017.06.30
21:1997.07.18
21:2017.06.30

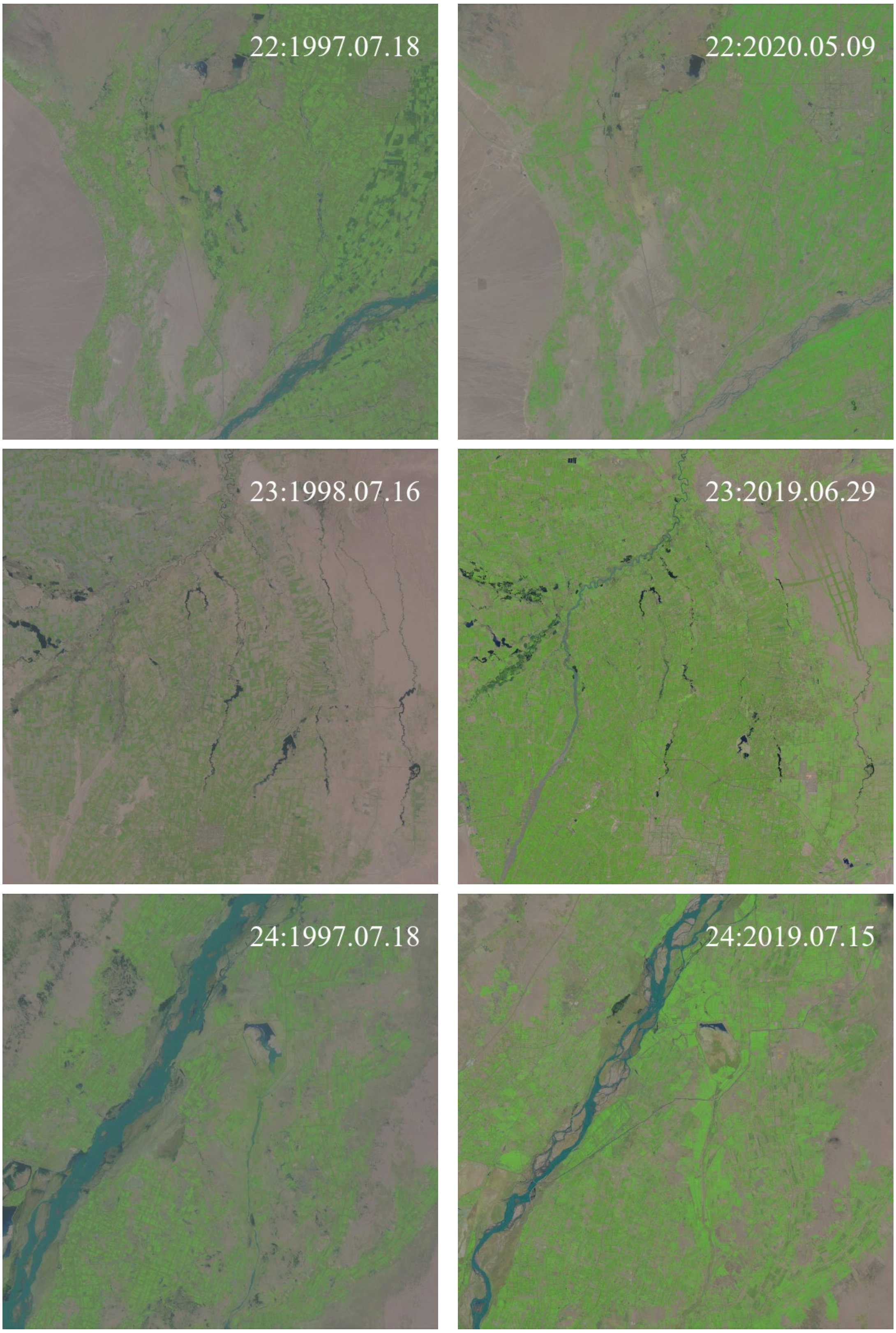
22:1997.07.18
22:2020.05.09
23:1998.07.16
23:2019.06.29
24:1997.07.18
24:2019.07.15

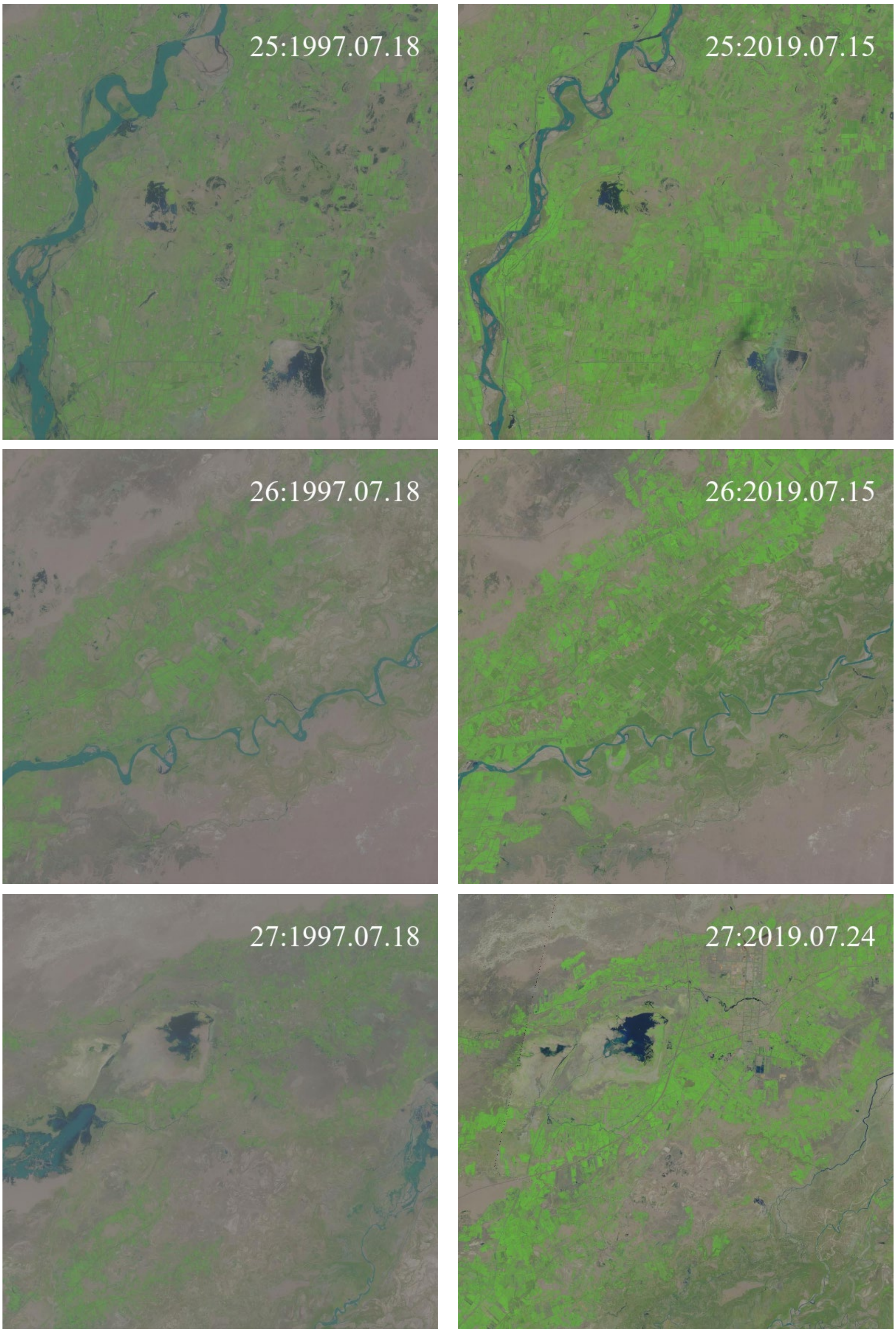
25:1997.07.18
25:2019.07.15
26:1997.07.18
26:2019.07.15
27:1997.07.18
27:2019.07.24

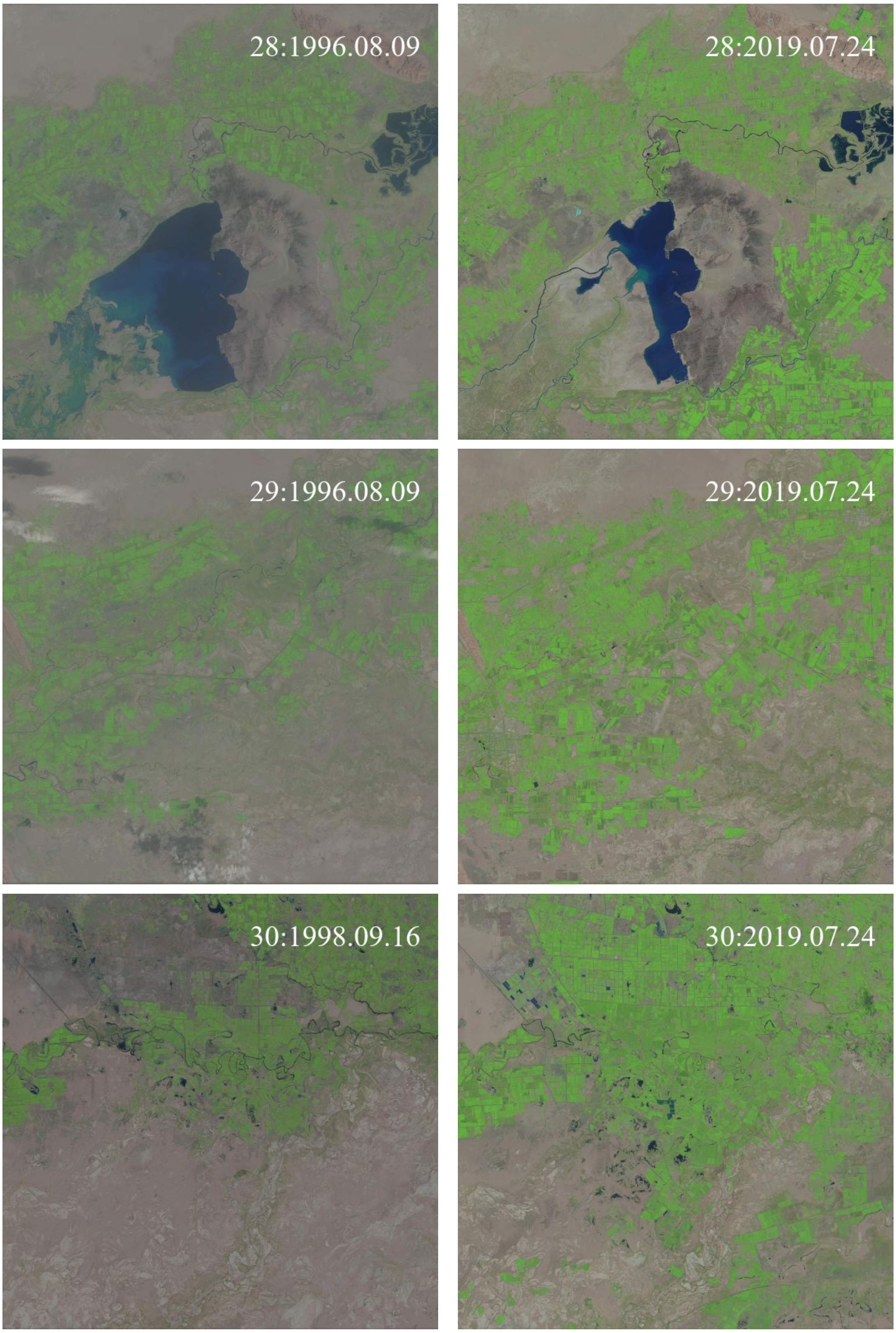
28:1996.08.09
28:2019.07.24
29:1996.08.09
29:2019.07.24
30:1998.09.16
30:2019.07.24

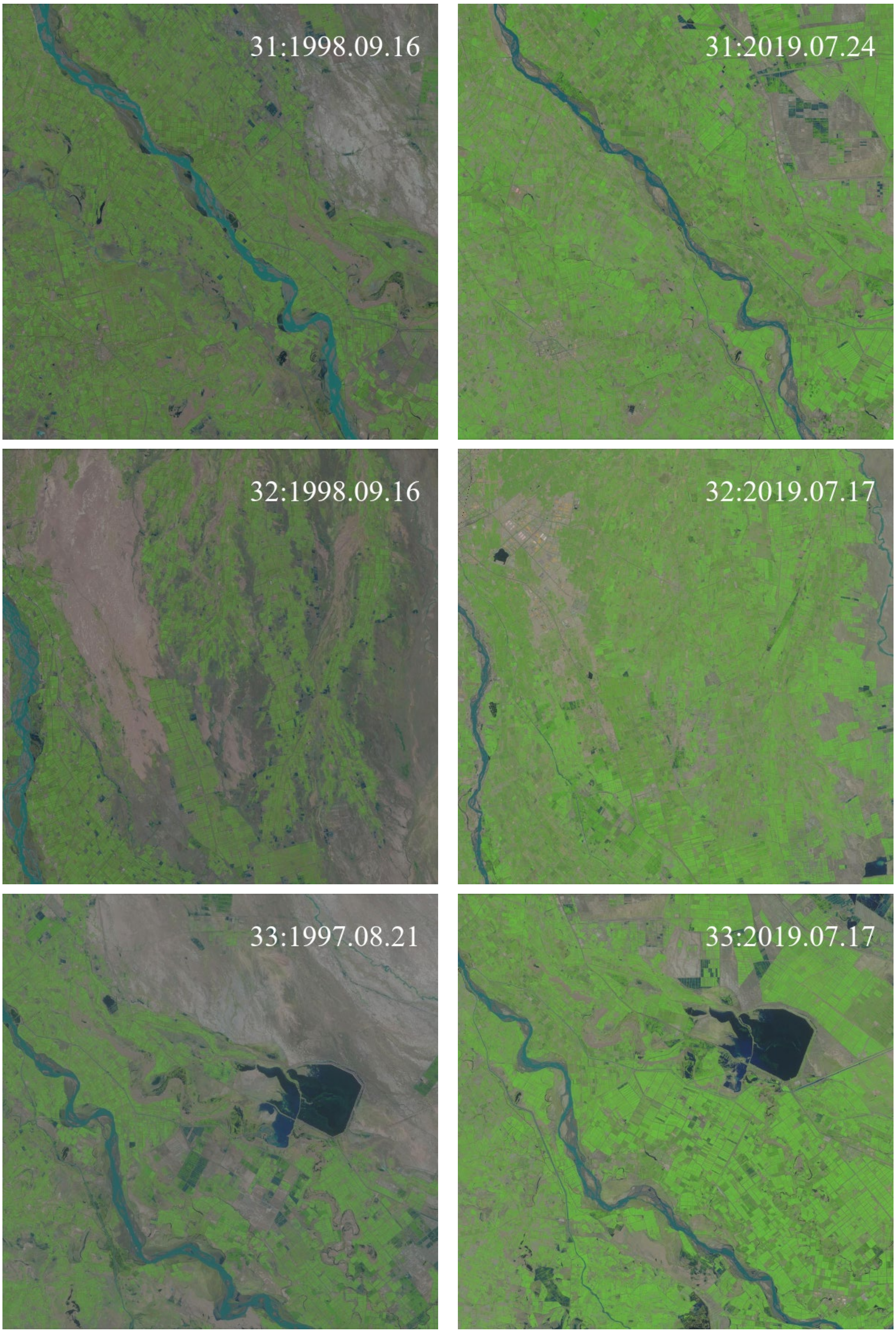
31:1998.09.16
31:2019.07.24
32:1998.09.16
32:2019.07.17
33:1997.08.21
33:2019.07.17

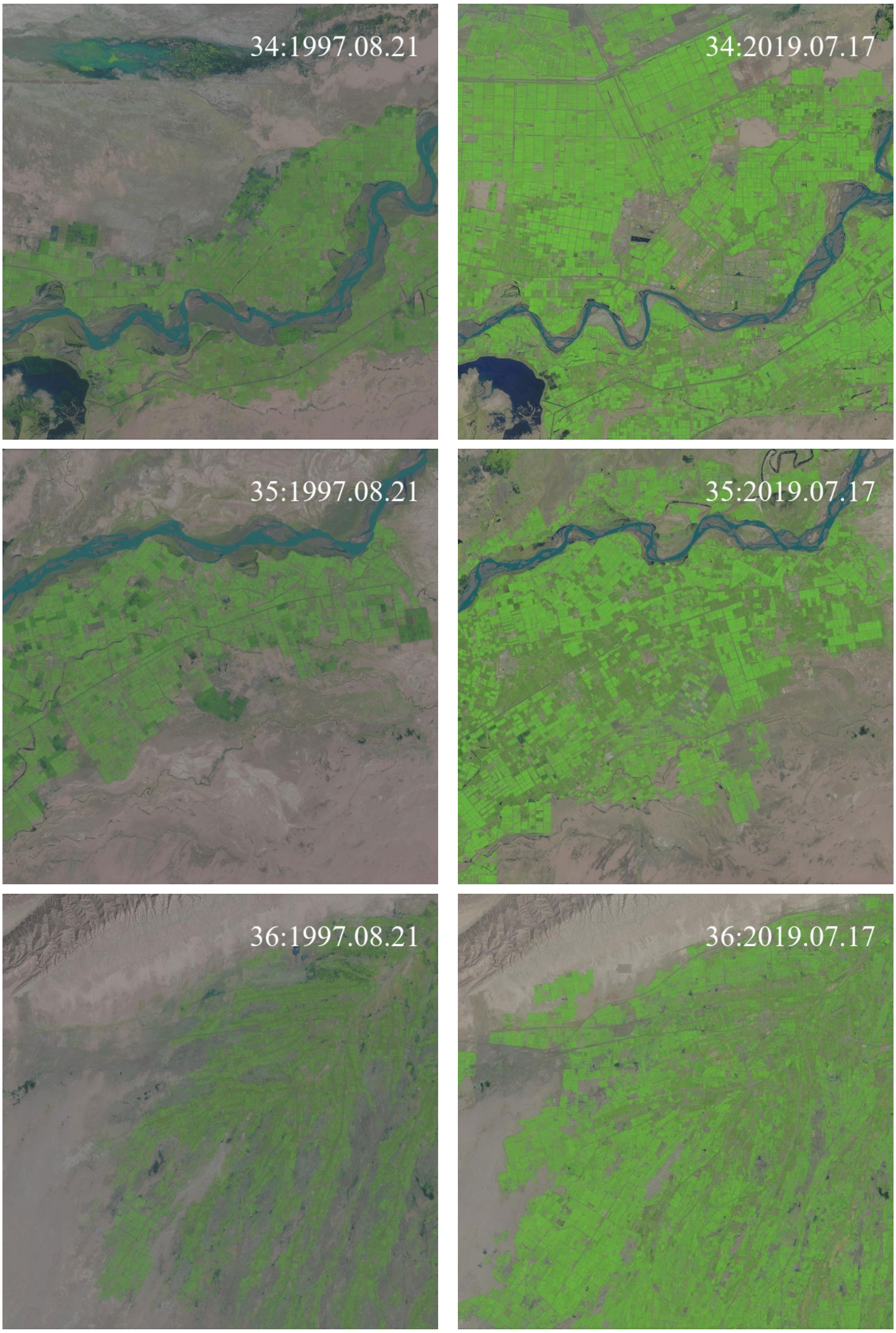
34:1997.08.21
34:2019.07.17
35:1997.08.21
35:2019.07.17
36:1997.08.21
36:2019.07.17

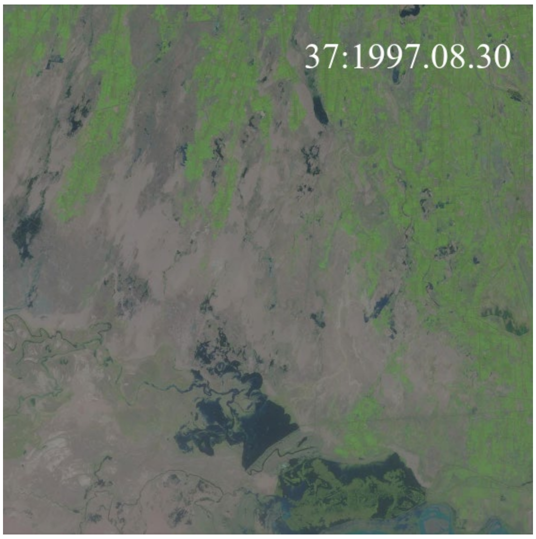
37:1997.08.30
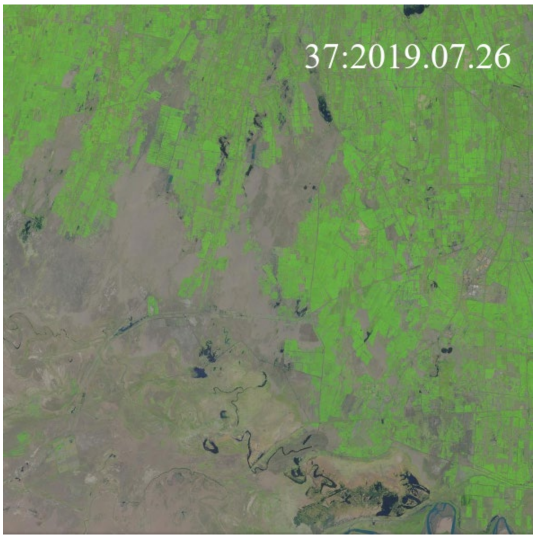
37:2019.07.26
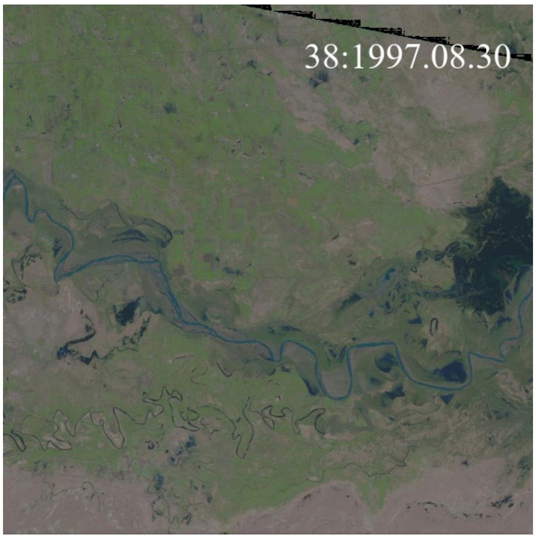
38:1997.08.30
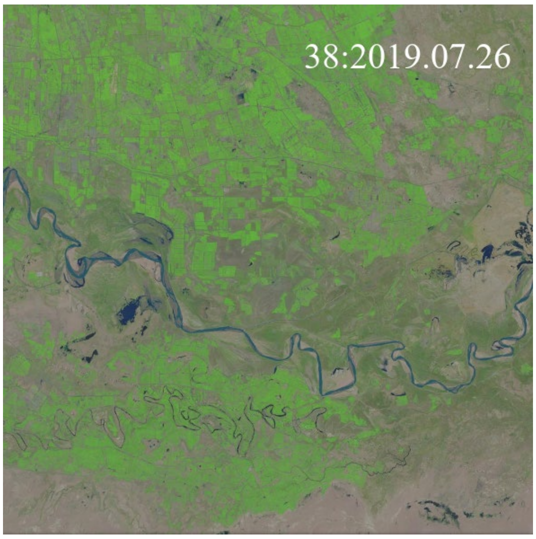
38:2019.07.26
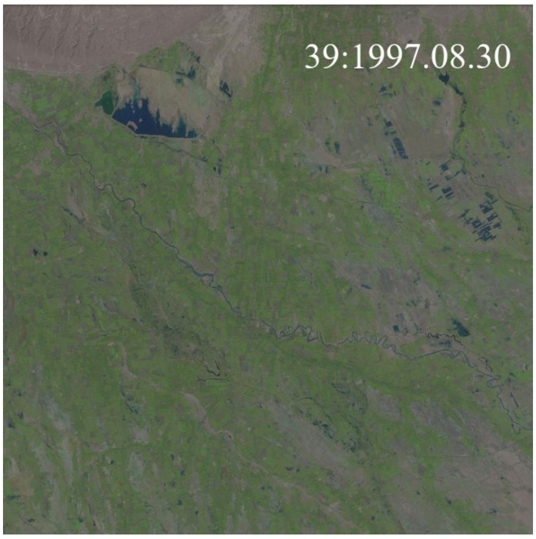
39:1997.08.30
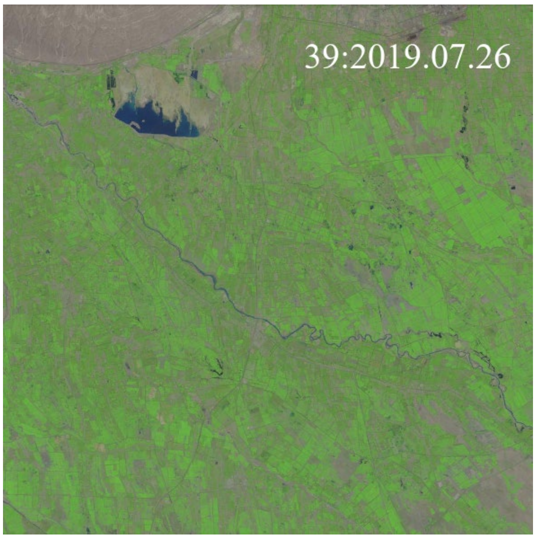
39:2019.07.26

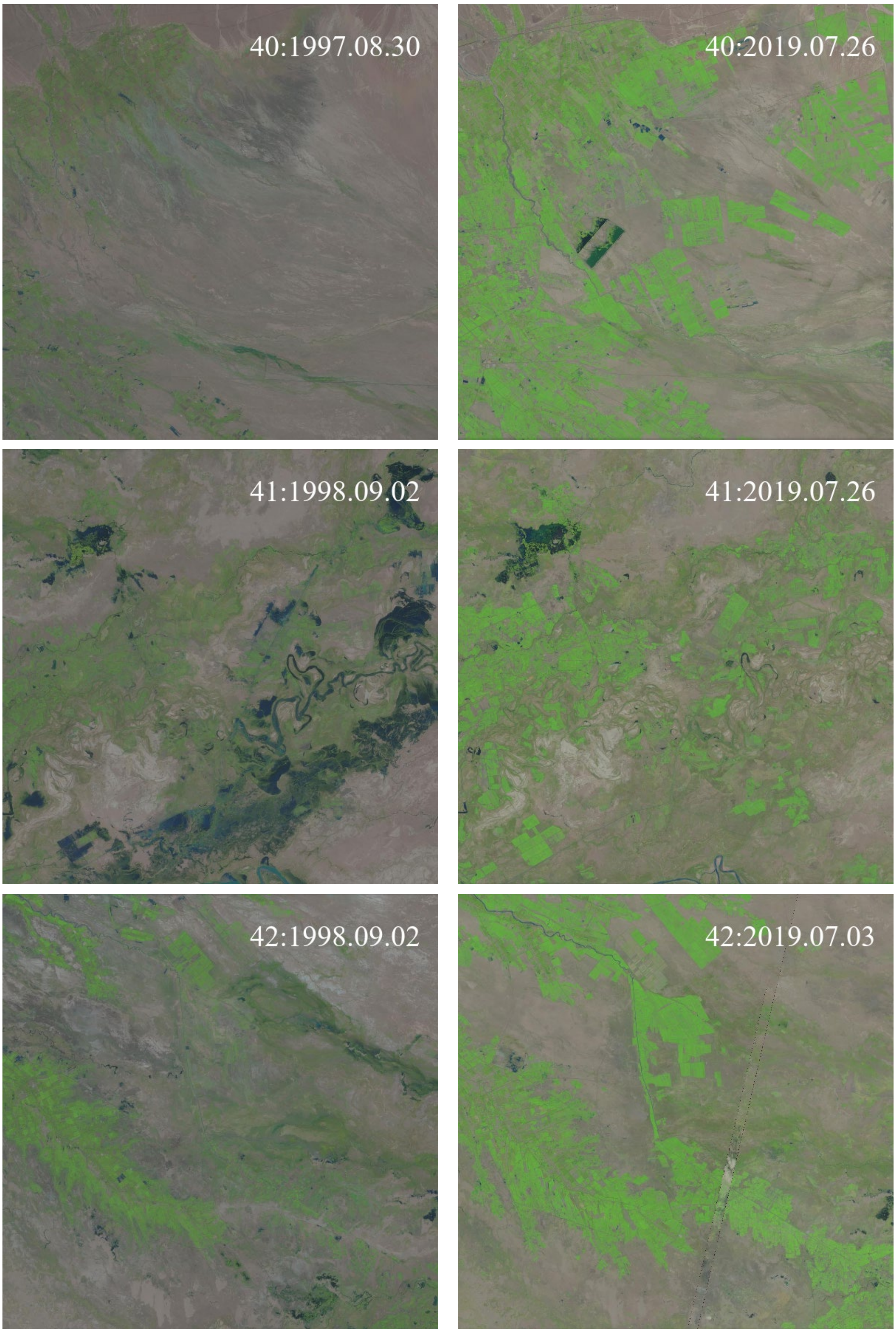
40:1997.08.30
40:2019.07.26
41:1998.09.02
41:2019.07.26
42:1998.09.02
42:2019.07.03

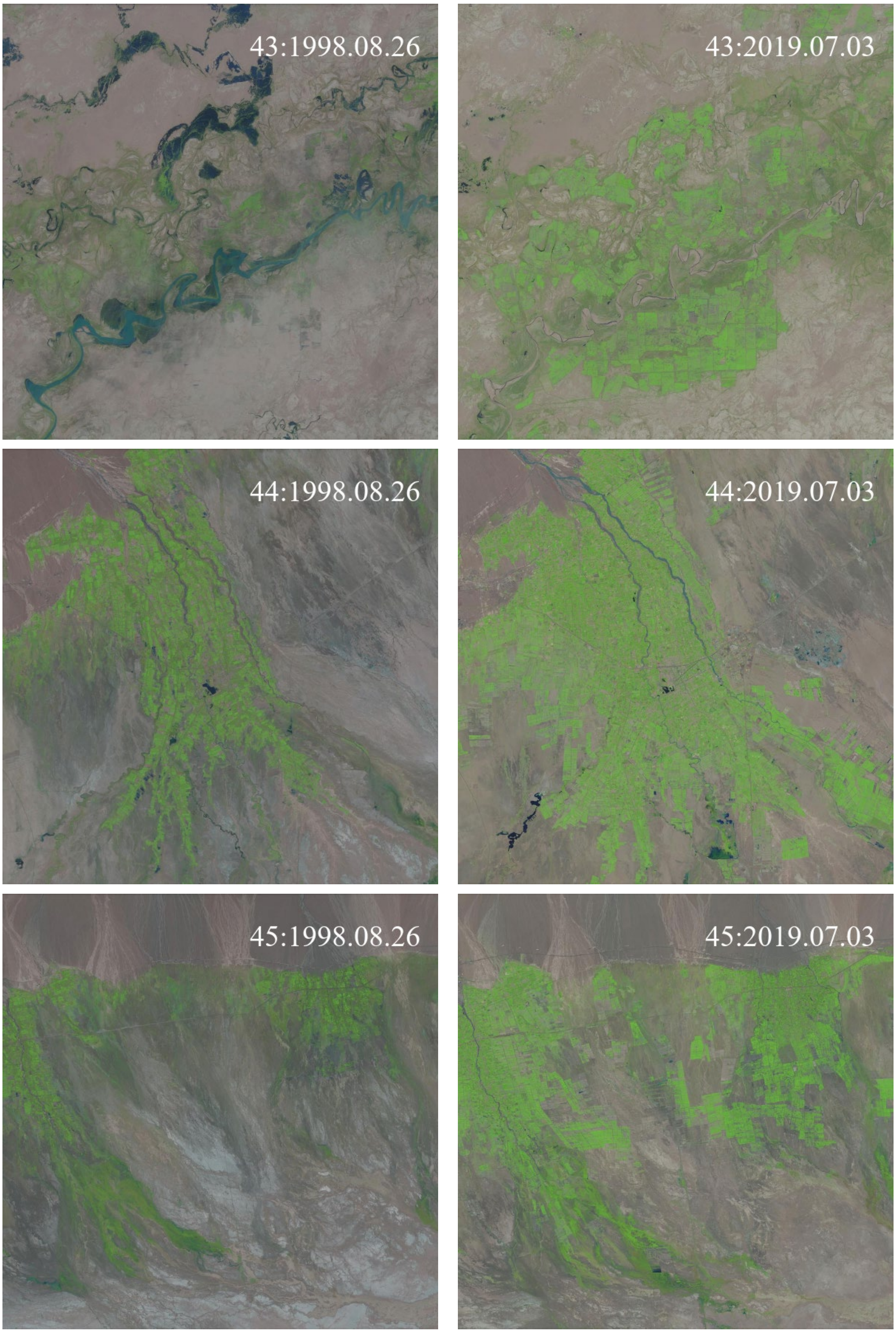
43:1998.08.26
43:2019.07.03
44:1998.08.26
44:2019.07.03
45:1998.08.26
45:2019.07.03

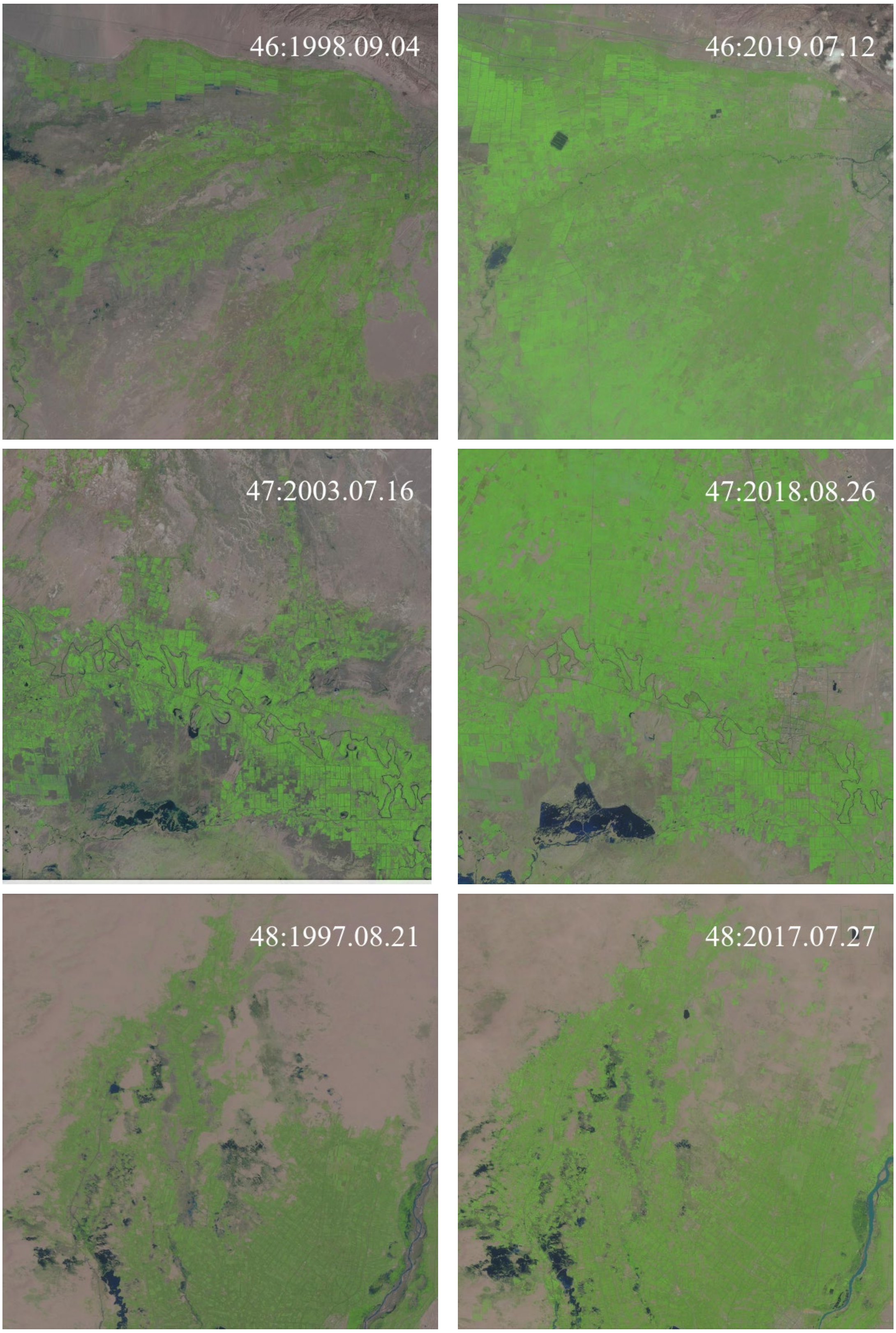
46:1998.09.04
46:2019.07.12
47:2003.07.16
47:2018.08.26
48:1997.08.21
48:2017.07.27

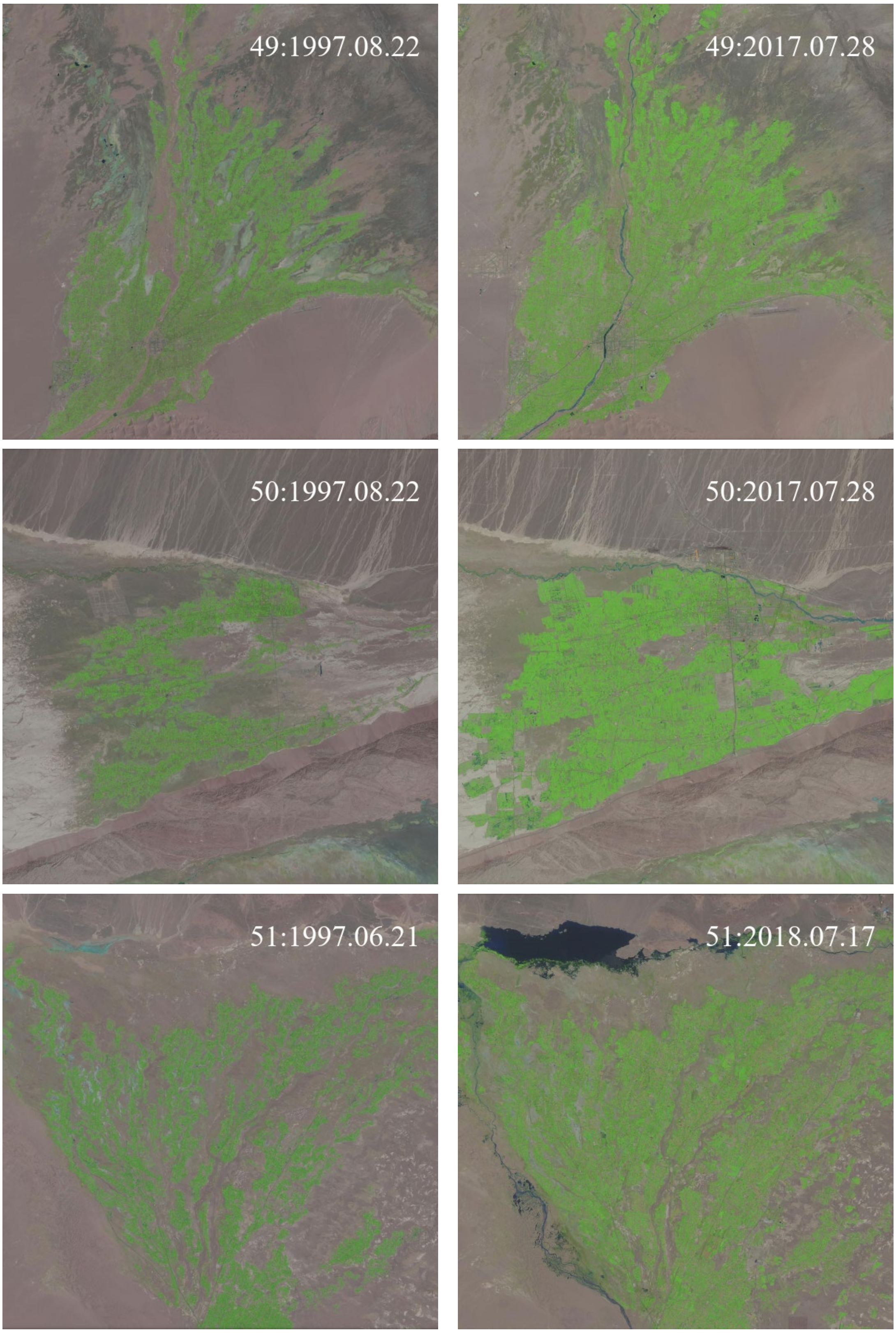
49:1997.08.22
49:2017.07.28
50:1997.08.22
50:2017.07.28
51:1997.06.21
51:2018.07.17

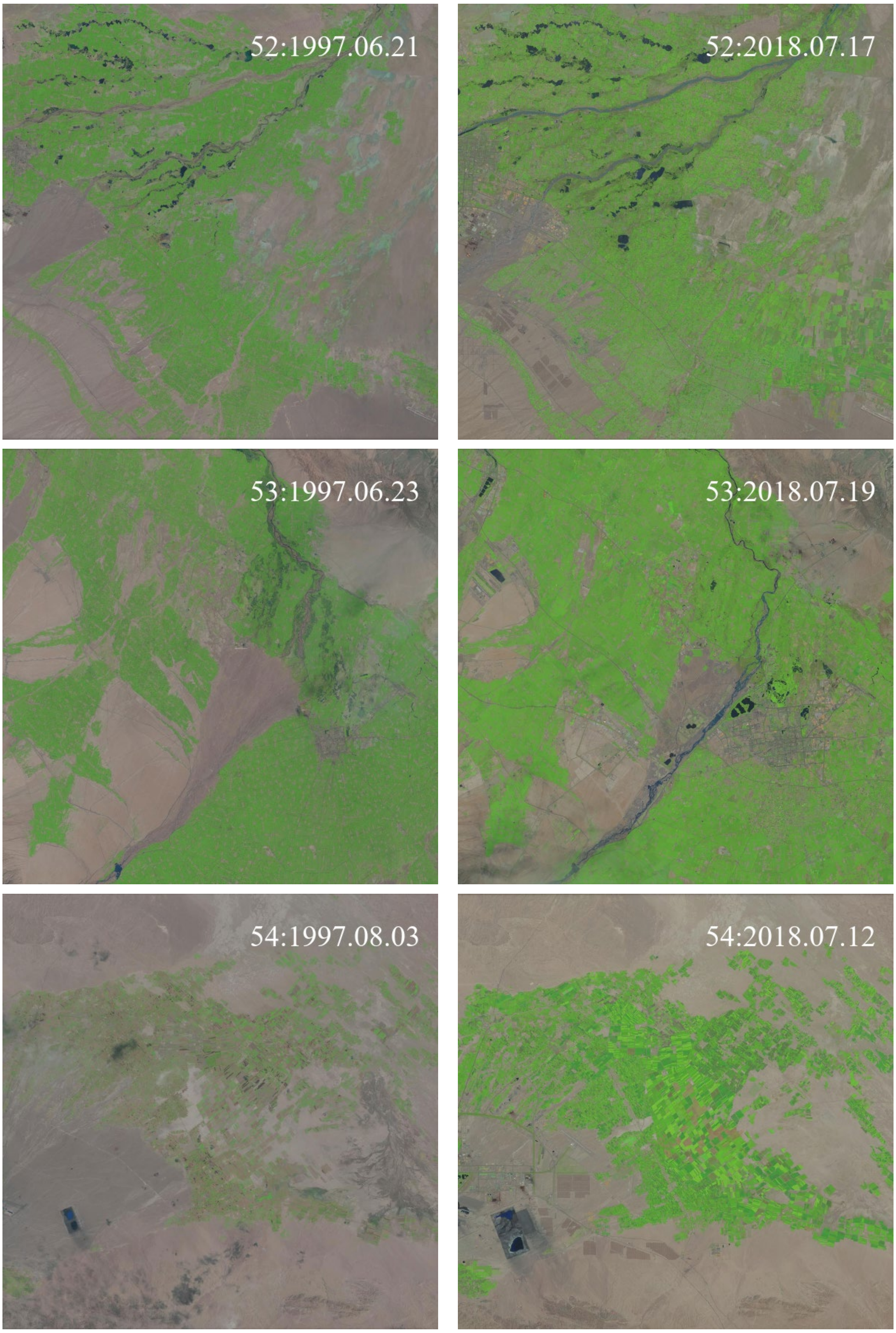
52:1997.06.21
52:2018.07.17
53:1997.06.23
53:2018.07.19
54:1997.08.03
54:2018.07.12

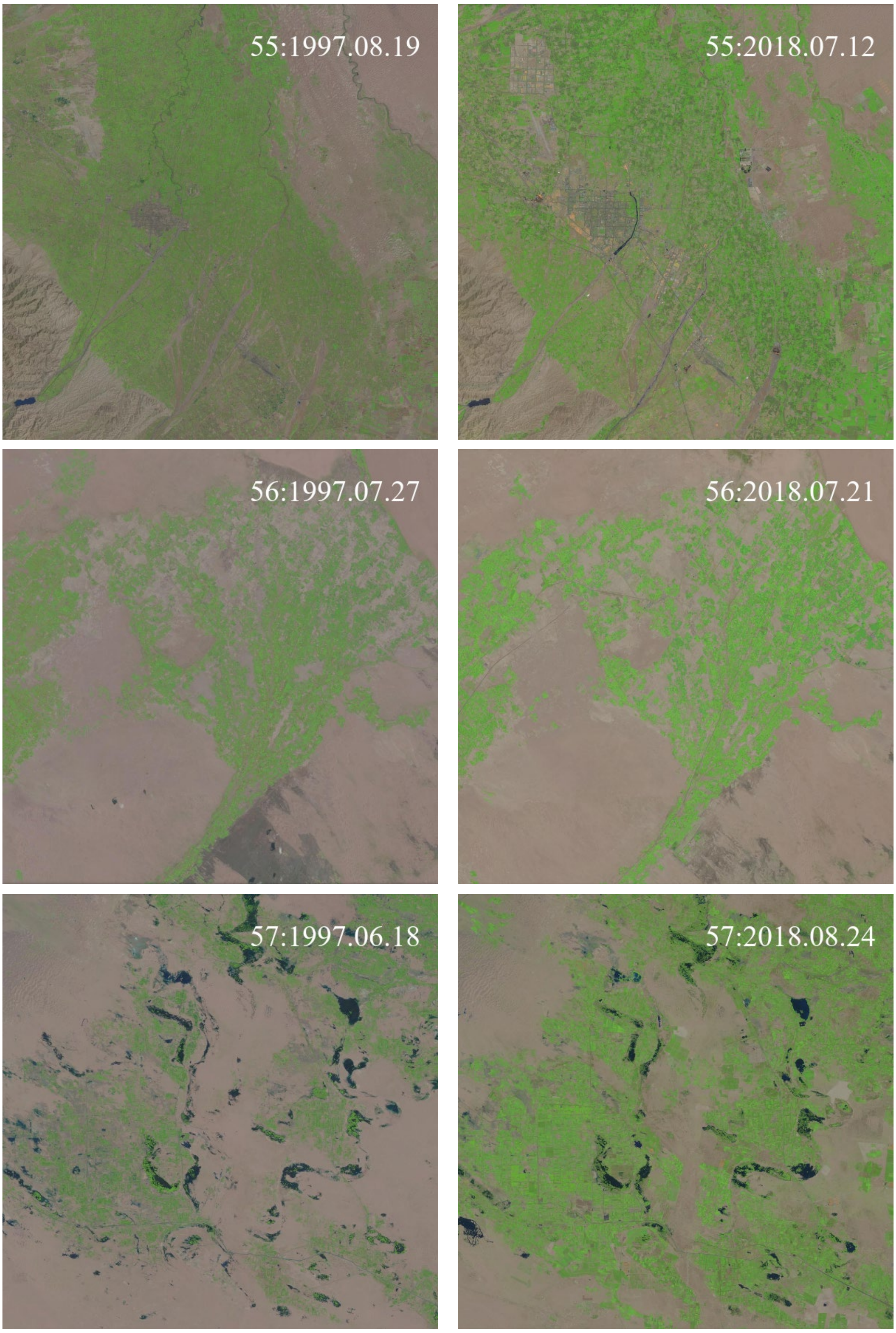

2. Sand stabilization measures

The greening areas of deserts due to sand stabilization measures were mainly

distributed in Kubuqi Desert (Number 58~62 in Fig S1). Images were from Google Earth for the resolution of the Landsat image is not fine enough.

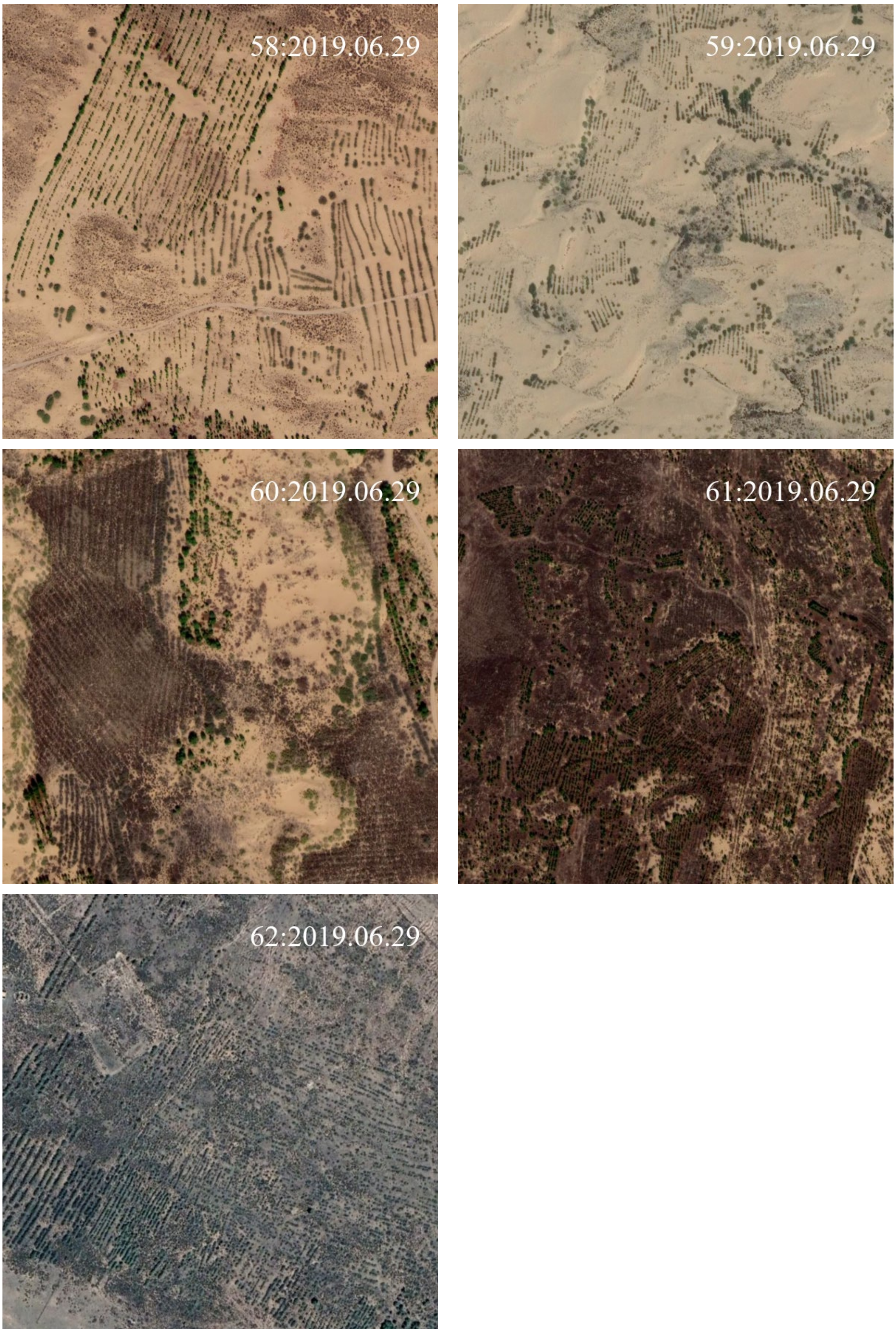

Supplement: S1 File — (PDF) [file pone.0256462.s001.pdf]
